# Chidamide enhances the sensitivity of gastric cancer to 5-fluorouracil chemotherapy by suppressing the HDAC3/HNF4A/TYMS axis

**DOI:** 10.1038/s41419-025-08247-y

**Published:** 2025-12-01

**Authors:** Xiaofei Zhang, Lei Shi, Yaping Gao, Chenyi Zhou, Xiyin Wang, Xiaonan Shi

**Affiliations:** 1 Department of Oncology, The First Affiliated Hospital of Zhengzhou University, Zhengzhou, Henan China; 2 Department of Urology, The First Affiliated Hospital of Zhengzhou University, Zhengzhou, Henan China

**Keywords:** Chemical biology, Diseases

## Abstract

Gastric cancer (GC) is among the most common malignant tumors in China and leads in incidence across all cancer types. For over three decades, the standard treatment has been traditional chemotherapy, which often involves monotherapy with 5-fluorouracil (5-FU) or its combination with other drugs. Unfortunately, nearly all cases of GC eventually develop resistance to 5-FU, typically displaying a median time to progression that ranges from 0 to 8 months. Therefore, elucidating the mechanisms of acquired resistance to 5-FU in GC continues to be a critical focus of ongoing research. Various gene and protein expression analyses were conducted utilizing techniques such as RT-qPCR, Western blot, IF, and IHC staining. Cell viability and proliferation were assessed using the CCK-8 assays and colony formation assays, respectively. Interactions among HDAC3, HNF4A, and TYMS were explored using ChIP, Co-IP, and dual-luciferase reporter assays. Chidamide increased the sensitivity of GC cells to 5-FU through the downregulation of TYMS and HDAC3. Additionally, the treatment with chidamide led to increased acetylation of HNF4A at lysine 458, due to the suppression of HDAC3, which in turn decreased phosphorylation of HNF4A at serine 313. Chidamide promoted the sensitivity of GC to 5-FU by suppressing the HDAC3/HNF4A/TYMS axis. This research may provide a foundation for using chidamide to counteract resistance to 5-FU in GC.

## Introduction

Gastric cancer (GC) ranks as one of the most prevalent malignant tumors in China, leading in incidence among all cancer types [1]. Annually, it accounts for approximately 783,000 deaths, nearly a quarter of all cancer fatalities [2]. Traditional chemotherapy, particularly monotherapy with 5-fluorouracil (5-FU) or its combination with other drugs, has been used as the standard treatment for over three decades [3]. Despite these efforts, the prognosis for patients with advanced GC remains dismal, with median overall survival rates barely exceeding one year [4]. Many patients initially respond to 5-FU but eventually relapse as their tumors develop resistance, leading to progression and death [4]. This resistance highlights the need for understanding the underlying mechanisms of tumor cell survival post-chemotherapy, a critical area of ongoing research.

Chidamide is the first orally available, subtype-selective histone deacetylase (HDAC) inhibitor approved in China [5]. It has shown a synergistic antitumor effect when combined with 5-FU in other cancers, such as colon [6]. However, the specific impact of chidamide on 5-FU resistance in GC treatment remains unexplored.

5-FU, structurally similar to thymine, is metabolized into fluorodeoxyuridine monophosphate (FdUMP) and fluorouridine triphosphate (FUTP) to exert its therapeutic effects [7]. FdUMP forms a crucial covalent complex with thymidylate synthase (TYMS) and 5,10-methylenetetrahydrofolate, further inhibiting TYMS and disrupting DNA synthesis [8]. Additionally, the misincorporation of FUTP during RNA synthesis impairs RNA function and hinders tumor cell proliferation [9]. This mechanism is essential for 5-FU’s cytotoxic and antitumor effects [9]. Preliminary research analyzing TYMS expression in GC using the TCGA database revealed high levels of TYMS expression in GC, highlighting its potential as a therapeutic target to enhance the efficacy of 5-FU against GC. Nevertheless, whether chidamide affects the downregulation of TYMS in the context of 5-FU treatment remains to be elucidated.

Chidamide functions as an inhibitor of Class I histone deacetylase (HDAC1/2/3) and Class IIb HDAC10 [10]. Our analysis of HDAC1/2/3/10 expression using the TCGA-STAD database revealed elevated levels of HDAC3 in GC. Furthermore, our preliminary study indicated a positive interaction between HDAC3 and TYMS expression in GC. HDAC3 has emerged as a promising therapeutic target for the treatment and prevention of GC [11]. For instance, inhibiting HDAC3 has been proposed to mitigate the development of GC [12]. However, whether HDAC3 was involved in chidamide-mediated TYMS remains to be investigated. In this study, we speculated that in GC, the inhibition of HDAC3 by chidamide might lead to a downregulation of TYMS expression, thereby enhancing the anti-tumor efficacy of 5-FU.

Hepatocyte nuclear factor 4 alpha (HNF4A), a member of the nuclear receptor family, has been implicated in various aspects of cancer development and progression [13]. It was also identified as a key oncogene in GC and considered a potential therapeutic target, particularly in early-stage GC [14]. Using the JASPAR database, we found that HNF4A bound to the promoter of *TYMS* and exhibited high expression in GC, with a positive correlation between HNF4A and TYMS expression. Studies indicated that HNF4A acts as a transcriptional regulator, facilitating the recruitment of RNA polymerase II to the promoters of target genes [15]. Therefore, we speculated that in GC, HNF4A may transcriptionally activate TYMS, thereby promoting its expression. However, whether the inhibition of HDAC3 by chidamide influences TYMS expression through HNF4A regulation remains to be elucidated.

The transcriptional regulatory functions of HNF4A are modulated through a variety of post-translational modifications (PTMs), including phosphorylation, acetylation, ubiquitination, and SUMOylation, each catalyzed by specific enzymes [16]. Among these PTMs, phosphorylation is particularly common [16]. Upon analyzing HNF4A, we discovered that phosphorylation modifications play a crucial role in its DNA-binding activity. Additionally, several sites on HNF4A, such as serine 313 (S313), undergo phosphorylation. At the same time, acetylation at lysine 458 (K458) of HNF4A reduces its transcriptional activation.

Based on the findings above, we hypothesized that chidamide inhibits HDAC3, resulting in decreased deacetylation at HNF4A K458. This led to increased acetylation levels of HNF4A, and consequently, reduced phosphorylation at S313. The decrease in phosphorylation impaired HNF4A’s ability to regulate the transcription of TYMS, which in turn inhibited TYMS expression and enhanced the anti-tumor effects of 5-FU. This proposed mechanism highlights a novel pathway through which chidamide may potentiate the effects of 5-FU in the treatment of GC.

## Materials and methods

### Cell culture

GC cell lines MKN-45, HGC-27, NCI-N87, and AGS, the human gastric epithelial cell line GES-1, along with 293T cell line were acquired from the American Type Culture Collection (ATCC, Manassas, VA, USA). These cell lines were cultured in RPMI 1640 medium (Gibco, Grand Island, NY, USA) supplemented with 10% fetal bovine serum (FBS, Gibco), 100 U/mL penicillin, and 100 μg/mL streptomycin. MKN‑45 was STR‑verified (ANSI/ATCC ASN‑0002.1‑2021) and certified mycoplasma-free. HGC‑27 was tested negative for mycoplasma via PCR and luminescence assays, with STR authentication performed. AGS and NCI‑N87 have been authenticated by STR profiling and confirmed mycoplasma-free.

### Cell transfection

Transfection procedures were carried out in line with methodologies described in prior studies [17]. In short, GC or 293T cells were prepared with a density of 5 × 10^6^ cells per well and were plated one day before the transfection process. Short hairpin RNAs aimed at silencing TYMS (sh-TYMS, Thermo Fisher Scientific, Waltham, MA, USA), HDAC3 (sh-HDAC3, Thermo Fisher Scientific), HNF4A (sh-HNF4A, Sigma-Aldrich, St. Louis, MO, USA), and a control shRNA and then integrated into GV102 vectors. The sequence of the shRNAs is shown in Table 1. To achieve overexpression, the complete HDAC3 or HNF4A gene sequence was amplified using PCR techniques and subsequently cloned into the pcDNA3.1 vector (Promega, Madison, WI, USA), resulting in the creation of OE-HDAC3 or OE-HNF4A. These constructs were then introduced into GC or 293T cells (5 × 10^6^ cells per well) employing Lipofectamine 3000 (Invitrogen, CA, USA) as the transfection agent. Six hours post-transfection, the cells’ medium was replaced with a standard culture medium. The cells were then left for an additional 48 h before being used in vitro analysis.

**Table 1:** shRNA sequences were listed.

Name	Sequence
shTYMS-1	5′-CACCGGGATTCTCCACCAGAGAAGACGAATCTTCTCTGGTGGAGAATCCC -3′
shTYMS-2	5′-CACCGGATTCTCCACCAGAGAAGAACGAATTCTTCTCTGGTGGAGAATCC -3′
shTYMS-3	5′- CACCGCCCAGTTTATGGCTTCCAGTCGAAACTGGAAGCCATAAACTGGGC-3′
shHDAC3-1	5′-CACCGACCGTGGCCTATTTCTACGACGAATCGTAGAAATAGGCCACGGTC-3′
shHDAC3-2	5′-CACCGGTCCTGCATTACGGTCTCTACGAATAGAGACCGTAATGCAGGACC-3′
shHDAC3-3	5′-CACCGCATTACGGTCTCTATAAGAACGAATTCTTATAGAGACCGTAATGC-3′
shHNF4A	5′-CACCGCCACATGTACTCCTGCAGATTCGAAAATCTGCAGGAGTACATGTGG-3′

### Cell treatment

GC cells, either transfected or not as previously described, were seeded in 24-well plates at a density of 0.5 × 10^5^ cells for two days. Subsequently, the cells were exposed to different treatments. For cytidine treatment, the cells were treated with varying concentrations (0, 0.01, 0.03, 0.1, 0.3, 1.0, 3.0, 10, or 30 μmol/L) of chidamide for 24, 48, or 72 h. For 5-FU treatment, GC cells received doses (0, 5, 10, 25, 50, 100, 200 μg/mL) of 5-FU for the same duration. In combined treatments, GC cells were treated with 5 μg/mL of 5-FU or various concentrations of chidamide (0, 0.01, 0.03, 0.1, 0.3, 1.0, 3.0, 10, or 30 μmol/L) for 48 h. For PKCi treatment, 500 nM of PKCi was added to the GC cell culture medium for one day. For λ-phosphatase, the GC cells were treated with λ-phosphatase (500U) for 30 min at 37 C. Afterward, the cells were collected for further analysis.

### Calculation of synergy scores for drug combinations

To quantitatively evaluate the pharmacological interaction between chidamide and 5-FU, the zero interaction potency (ZIP) synergy scores were calculated according to the SynergyFinder [18]. Cell viability was assessed using the cell counting Kit-8 (CCK-8) assay after treating gastric cancer cells (MKN-45, HGC-27, and NCI-N87) with chidamide, 5-FU, or their combinations at various concentrations for 24 and 48 h. ZIP values were calculated for combinations administered at a constant molar ratio, with ZIP > 10 indicating synergism, ZIP = −10–10 indicating an additive effect, and ZIP < −10 indicating antagonism.

### CCK-8 assay and IC₅₀ determination

Cell viability was evaluated using the CCK-8 assay kit (Boster Biological Technology Co., Ltd, China). Briefly, 10 µL of CCK-8 solution was added to each well containing GC cells that were treated as mentioned above, followed by a 2-h incubation. The absorbance at 450 nm was then measured using a microplate reader (Thermo Fisher Scientific).

To calculate the half-maximal inhibitory concentration (IC₅₀) of 5-FU, GC cells were treated with the indicated concentrations of 5-FU for 24, 48, or 72 h. Cell viability data were analyzed using GraphPad Prism software with a nonlinear regression model (four-parameter logistic curve) to determine IC₅₀ values.

### Colony formation assay

GC cells were cultured in 6-well plates at a density of 1 × 10^4^ cells per well. Twelve hours later, the cells underwent transfection or treatment as previously described and were then incubated for an additional 7 days. Following this period, the cells were treated with 3.7% paraformaldehyde for 20 min, then rinsed twice with PBS, and stained with 10% crystal violet. Colony counts were performed using a microscope (Olympus, Tokyo, Japan).

### Detection of 5-FU resistance

GC cells were plated in 96-well plates at a density of 1 × 10^4^ cells per well and incubated overnight. The cells were then exposed to various concentrations of 5-FU (0, 5, 10, 25, 50, 100, 200 μg/mL) in a medium containing 10% FBS for 24 h. Following treatment, 10 µL of CCK-8 solution was pipetted into each well and incubated for an additional 2 h. The absorbance at 450 nm was then measured using a microplate reader (Thermo Fisher Scientific). The half-maximal inhibitory concentration (IC50) of the 5-FU treated samples was determined using GraphPad Prism version 5.0 software.

### Animal studies

The experimental protocols were sanctioned by the pertinent local regulatory bodies. Male BALB/c nude mice, aged 5–6 weeks, were procured from Hunan STJ Laboratory Animal Co., Ltd., Hunan, China. The animals were maintained under controlled environmental conditions, with humidity maintained at 55% ± 10%, a 12-h light/dark cycle, and a stable temperature of 23 ± 2 °C. To establish a murine tumor model, HGC-27 cells or MKN-45 cells were administered subcutaneously at a dosage of 5 × 10^6 cells/kg on the right side of the dorsal midline of the mice. Afterward, the mice were allocated into four experimental groups: PBS only, 100 mg/kg 5-FU only, 25 mg/kg/day chidamide only, and a combination treatment of 50 mg/kg 5-FU with 12.5 mg/kg/day chidamide, with each group comprising six mice. Chidamide was administered daily via gastric gavage, while 5-FU was injected intraperitoneally every three days. Both body weight and tumor volume were measured at five-day intervals. The study concluded 25 days after the initiation of treatments. All experimental methods were performed in strict accordance with guidelines provided by the The First Affiliated Hospital of Zhengzhou University [2024-KY-2002-003], and adhered to the American Physiological Society’s “Guides for the Care and Use of Laboratory Animals”. Although no formal statistical methods were used to calculate sample size, the group size (*n* = 6) was based on previous studies using similar in vivo models and was considered sufficient to detect biologically relevant differences in tumor growth. Animals were randomly assigned to treatment groups. Investigators were not blinded to group allocation during the experiments and outcome assessment.

### Dual-luciferase reporter assay

The 293T cells were transfected with OE-HDAC3, OE-HNF4A, or their respective negative controls using Lipofectamine 3000 transfection reagent (Invitrogen). After a 48-h incubation, luciferase activities were quantified utilizing the Dual-Luciferase Reporter Assay Kit (Promega).

### Chromatin immunoprecipitation (ChIP) assay

GC cells were transfected as described previously, harvested, and treated with ChIP lysis buffer (Merck Millipore, MA, USA). After cell cross-linking and sonication, the samples were centrifuged, and the supernatant was transferred to a new tube. To preclear the lysates, they were incubated with protein A/G Sepharose for 2 h at 4 °C, then centrifuged to collect the supernatant. Next, 1 μL of primary antibodies, or IgG antibody (ab205718, 1:1000, Abcam) was added to the samples along with 20 μL of 50×PIC (protease inhibitor cocktail), 900 μL of ChIP Dilution Buffer. Post-incubation, the beads were washed several times with sonication buffer, wash buffers, and TE buffers, and then eluted by heating with Elution Buffer at 65 °C for 10 min. After centrifugation, 21 μL of NaCl was added and the mixture was incubated again at 65 °C overnight to de-crosslink. RNase A was added post-de-crosslinking to remove RNA contaminants, followed by incubation at 37 °C for 1 h. The samples were then treated with 20 μL of 1 M Tris-HCl, 10 μL of 0.5 M EDTA, and 2 μL of 10 mg/mL proteinase K and incubated at 45 °C for 2 h. Finally, DNA was extracted, purified, and quantified using agarose gel electrophoresis.

The ChIP assay was conducted using the EZ-ChIP kit (Upstate, Lake Placid, NY, USA). Initially, GC cells, previously transfected as described, were treated with 0.75% formaldehyde at room temperature for 10 min, followed by washing with PBS. Cells were then resuspended in SDS lysis buffer and the chromatin was sheared by sonication. Chromatin fragments were immunoprecipitated using H3K27Ac antibody (ab4729, 1:200, Abcam, Cambridge, UK), anti-HNF4A antibody (1:1000, ab181604, Abcam), anti-HDAC3 antibody (1:200, ab137704, Abcam), or anti-IgG antibody (1:2000, ab205718, Abcam). The DNA recovered from these immunoprecipitations was subsequently analyzed by polymerase chain reaction (PCR).

### Real-time polymerase chain reaction (RT-qPCR)

Total RNA was extracted from cells or tissues utilizing the Trizol reagent (Beyotime, Shanghai, China). One microgram of total RNA was reverse transcribed using SYBR Premix Ex Taq (Takara, Dalian, China) following the manufacturer’s instructions. Quantitative PCR was conducted with the TaqMan® Universal PCR Master Mix (4305719, Thermo Fisher Scientific). The primers, provided by Origene Biotech (Wuxi, Jiangsu, China), were as follows:

TYMS forward: 5´- GGTGTTTTGGAGGAGTTGCTGTG-3´, reverse 5´- GGAGAATCCCAGGCTGTCCAAA-3´; DHFR forward: 5´- CCCATCACATGTGGCACTCT-3´, reverse 5´-GACTATGTTCCGCCCACACA-3´; DPYS forward: 5´- GCACTTTCAACACCTGCCAG-3´, reverse 5´-TTCAGTGTGGCGACTTCTCC-3´; DTYMK forward: 5´- CCTCGTCGTGGACAGATACG-3´, reverse 5´-GCTCTGCTCATGTCCACACT -3´; DUT forward: 5´- GAAATGACTCCCCTCTGCCC-3´, reverse 5´- AAGTGTTTTGCAGCCAAGCC-3´; FPGS forward: 5´- CAGTGACACTGGACCAGGTC -3´, reverse 5´-GGAAGGAGAGACCAAGGCAC-3´; GGH forward: 5´- CTGCAGGTGCGAGAGTTGTA-3´, reverse 5´- AGGTGCTTTCTCTGGATGCC-3´; NME1 forward: 5´- GAGCAGAAAGGATTCCGCCT -3´, reverse 5´- CAGGGAGAACTCACAGCTCC-3´; NT5C forward: 5´- AGTGGCCAGTGTGTACGAAG -3´, reverse 5´- GTATCTGCCTGCTCCACTCC-3´; RRM1 forward: 5´- GGTCTGGATGAGGTTTGGGG -3´, reverse 5´- CTGGGCTTCTGCACTCTCAA-3´; UCK2 forward: 5´- TGAGGTGGACTATCGCCAGA-3´, reverse 5´- ATGAGGTTGATGGCCACCAG-3´; UNG forward: 5´- CTCATAAGGAGCGAGGCTGG-3´, reverse 5´- TGAAAGGTGCACATCTCCCC-3´; UMPS forward: 5´- GTAGAGACGGTGGAGGAGGT -3´, reverse 5´- TGGTGGAGCCCAGTTTTTGT-3´; GAPDH forward: 5´- GTCTCCTCTGACTTCAACAGCG-3´, reverse 5´- ACCACCCTGTTGCTGTA GCCAA-3´. Relative mRNA expression levels were determined using the 2^-ΔΔCt^ method and normalized to GAPDH expression.

### Co-immunoprecipitation (Co-IP) assay

Site-directed mutagenesis was performed to generate the acetylation-deficient mutant HNF4A(K458R) with the Fast Mutagenesis System Kit (FM111-01, TransGen Biotech, Beijing, China,), following the manufacturer’s protocol. Primers were designed to introduce the specific point mutation and to incorporate a C-terminal Flag tag into both the wild-type and mutant constructs to facilitate downstream detection. All plasmid constructs were verified by Sanger sequencing (Tsingke Biotechnology, Beijing, China) to confirm the presence of the desired mutations and the integrity of the coding sequences. 293T cells were then transfected with either HA-tagged or Myc-tagged HNF4A, Flag-HNF4A-WT or Flag-HNF4A-K458R plasmids. Twenty-four hours after transfection, cells were treated with chidamide 30 µM for 48 h. Similarly, MKN45 and NCI-H87 cells were transfected with the same plasmids using Lipofectamine 3000. In some groups, HNF4A knockdown was performed using specific sh-RNA constructs prior to plasmid transfection. Chidamide treatment was conducted under the same conditions as in 293T cells. Afterward, the supernatant was collected, and non-specific binders were removed by incubating with protein G-plus beads (Santa Cruz, CA, USA) for 30 min at 4 °C. After centrifugation at 1000 × *g* for 5 min at 4 °C, the supernatant was cleared and incubated with an anti-HA tag antibody (ab236632, 1:30, Abcam) or anti-Myc tag antibody (ab32, 1:50, Abcam) for 1 h at 4 °C on a rotating platform. Protein G-plus beads were then added to the mixture, which was incubated overnight at 4 °C with continuous rotation. The following day, the beads were washed four times with ice-cold 0.2% digitonin wash buffer and twice with ice-cold PBS. For immunoblot analysis, proteins were eluted from the beads by boiling in a twofold concentrated protein sample buffer for 3 min. Control antibodies were also used.

GC cells were transfected without or with either Flag-HNF4A(WT), Flag-HNF4A(K458R) constructs or Flag-HDAC3 plasmids and treated with or without the selective HDAC3 inhibitor RGFP966 (1 µM) for 20 h [19] or AMPK inhibitor BAY-3827 (5 μM) or vehicle (DMSO) for 24 h [20]. Afterwards, the cells were washed twice with PBS and then treated with RIP lysis buffer (Merck Millipore). The resulting cell lysates were sonicated on ice using an IP buffer and centrifuged at 12,000 rpm for 10 min. A 30 μL aliquot of the supernatant was collected as the Input sample. Additionally, 420 μL of the supernatant was subjected to overnight immunoprecipitation at 4 °C using an anti-HDAC3 antibody (1:50, ab32369, Abcam) or a control nonspecific IgG antibody (1:1000, ab18413, Abcam). Protein A was added, and the mixture was incubated for 1 h at 4°C. Following four washes with IP buffer, the sample was centrifuged for 2 min, and the supernatant was discarded. Subsequently, 30 μL of 2X SDS lysate was added and incubated for 10 min. The samples were then processed for immunoblotting with anti-HDAC3 or HNF4A primary antibodies.

### Western blot

Protein lysates were prepared using radio-immunoprecipitation assay (RIPA) buffer with protease inhibitors and incubated at 4 °C for 30 min for complete lysis (Beyotime Inc., Haimen, Jiangsu, China). Protein concentrations were determined using the BCA Protein Assay Kit (10741395, Thermo Fisher Scientific). Thirty micrograms of protein from each lysate were subjected to SDS-PAGE and transferred onto polyvinylidene fluoride (PVDF) membranes (Merck Millipore). The membranes were blocked and incubated with primary antibodies: TYMS (ab108995, 1:1000), HDAC2 (ab32117, 1:2000), HDAC1 (ab109411, 1:1000), HDAC10 (ab108934, 1:1000), HDAC3 (ab32369, 1:5000), HNF4A (ab181604, 1:1000), HNF4A phospho S313 (ab78356, 1:1000), Acetyl-HNF4A (Ac-K, orb676366, 1:1000, Biorbyt, Hefei, China), anti-O-GlcNAc (ab2739, 1:1000), anti-phosphoserine (ab9332, 1:1000), Histone H3 acetyl K27 (ab4729, 1:1000), Histone H3 acetyl K18 (ab40888, 1:2000), Histone H3 acetyl K14 (ab52946, 1:2000), Histone H3 acetyl K9 (ab32129, 1:500), Histone H4 acetyl K8 (ab45166, 1:5000), Histone H3 (ab1791, 1:2000), Histone H4 (ab177840, 1:1000), Acetyl-HNF4A (orb676366, 1:1000; biorbyt, Huhan, Hefei, China), and β-actin (ab8226, 1:1000, Abcam) as a loading control. After PBS washing, membranes were incubated with secondary antibodies (ab7090, 1:500 or ab150165, 1:500, both Abcam). Protein bands were visualized using an enhanced chemiluminescence (ECL) system (WBULS0100, Merck Millipore). Full, uncropped Western blot membranes are provided in the Supplementary Material.

### GST-pulldown assay

Site-specific mutations were introduced into HDAC3 through PCR-based, oligonucleotide-directed mutagenesis. The resulting mutant cDNA ORF coding plasmids of HDAC3 were then integrated into a pcDNA3.1 vector using primers that added an HA-tag or Flag-tag at their C-terminal. To examine the interaction between various truncated forms of HDAC3 were purchased from Origene (Shanghai, China). HNF4A and the corresponding truncated forms of HNF4A were purchased from Origene. The DAD domain of HDAC3 was expressed as a GST fusion protein. The assays were conducted by incubating 5 µg of GST-tagged protein with 85 µL of 293T whole-cell lysate (prepared from one 100-mm dish lysed in 1 mL TAP buffer) in a total volume of 600 µL of TAP buffer (50 mM Tris-HCl, pH 7.5, 200 mM NaCl, 0.1% Triton X-100, 10% glycerol, with protease inhibitor cocktail). A 10% input sample (8.5 μL in 20 μL Laemmli sample buffer) was reserved. The mixture was incubated with rotation at 4 °C for 2–3 h, after which 25 μL of Glutathione-sepharose beads were added and incubated for an additional hour. The beads were then washed four times with 1 mL of TAP buffer and resuspended in 20 μL Laemmli sample buffer before analysis by SDS-PAGE and immunoblotting.

### Immunofluorescent (IF) staining

GC tissues were incubated in 3.7% buffered formalin and then embedded in paraffin. IF staining was carried out on 5 μm sections from these paraffin blocks. Sections were rehydrated and incubated in a blocking solution containing 1% bovine serum albumin for 1 h. They were then incubated at 4 °C overnight with anti-HDAC3 antibody (ab32369, 1:250, Abcam) and anti-HNF4A antibody (ab201460, 1:2000, Abcam). The next day, sections were thoroughly washed with PBS and incubated with secondary antibodies (Abcam) for 2 h at room temperature. Nuclear staining was performed using DAPI (Life Technologies, Waltham, CA, USA). The fluorescently labeled cells were examined using a ZEISS microscope.

### Immunohistochemistry (IHC) staining

GC tissues were fixed in 3.7% buffered formalin and subsequently embedded in paraffin. IHC staining was conducted on 5 µm thick sections prepared from the paraffin blocks. The sections were incubated with primary antibodies, including Ki67 (ab15580, 1:1000, Abcam), HDAC3 (ab32369, 1:250, Abcam), HNF4A (ab201460, 1:2000, Abcam), and TYMS (ab108995, 1:100, Abcam) for 1 h at room temperature. After primary antibody incubation, sections were treated with appropriate secondary antibodies. Hematoxylin was used for counterstaining before images were captured using a Leica confocal microscope.

### Kaplan–Meier survival analysis

To evaluate the clinical relevance of HDAC3, HNF4A, and TYMS expression in gastric cancer, we performed overall survival analysis using the Kaplan–Meier Plotter database (https://kmplot.com/analysis). This platform integrates gene expression and survival data from multiple gastric cancer cohorts. The following probe sets were used for analysis: HDAC3 (216326_s_at), TYMS (217684_at), and HNF4A (208429_x_at). Patients were stratified into high and low expression groups based on the median expression of each gene. Survival curves were generated using the default settings, and log-rank *p*-values were calculated to assess statistical significance.

### Statistical analysis

Statistical analysis was conducted using SPSS version 20.0 (IBM, Armonk, NY, USA). Results are reported as mean ± standard deviation, derived from at least three independent experiments. Due to the small sample size (*n* = 3), formal tests for normality were not performed, as results from such tests are unreliable at this scale [21]. Parametric tests (t-test or ANOVA) were applied based on the assumption of approximate normal distribution—a common approach in exploratory biological studies where sample sizes are limited. Differences between two groups were assessed using unpaired Student’s *t* tests, while comparisons among more than two groups utilized one-way analysis of variance (ANOVA) with Tukey’s post hoc test for multiple comparisons. A *P* value of less than 0.05 was deemed statistically significant. Sample sizes were estimated based on preliminary data to ensure a statistical power of at least 80% for detecting pre-specified effect sizes at a significance level of 0.05.

## Results

### Chidamide enhances the sensitivity of gastric cancer cells to 5-FU

**Fig. 1:**
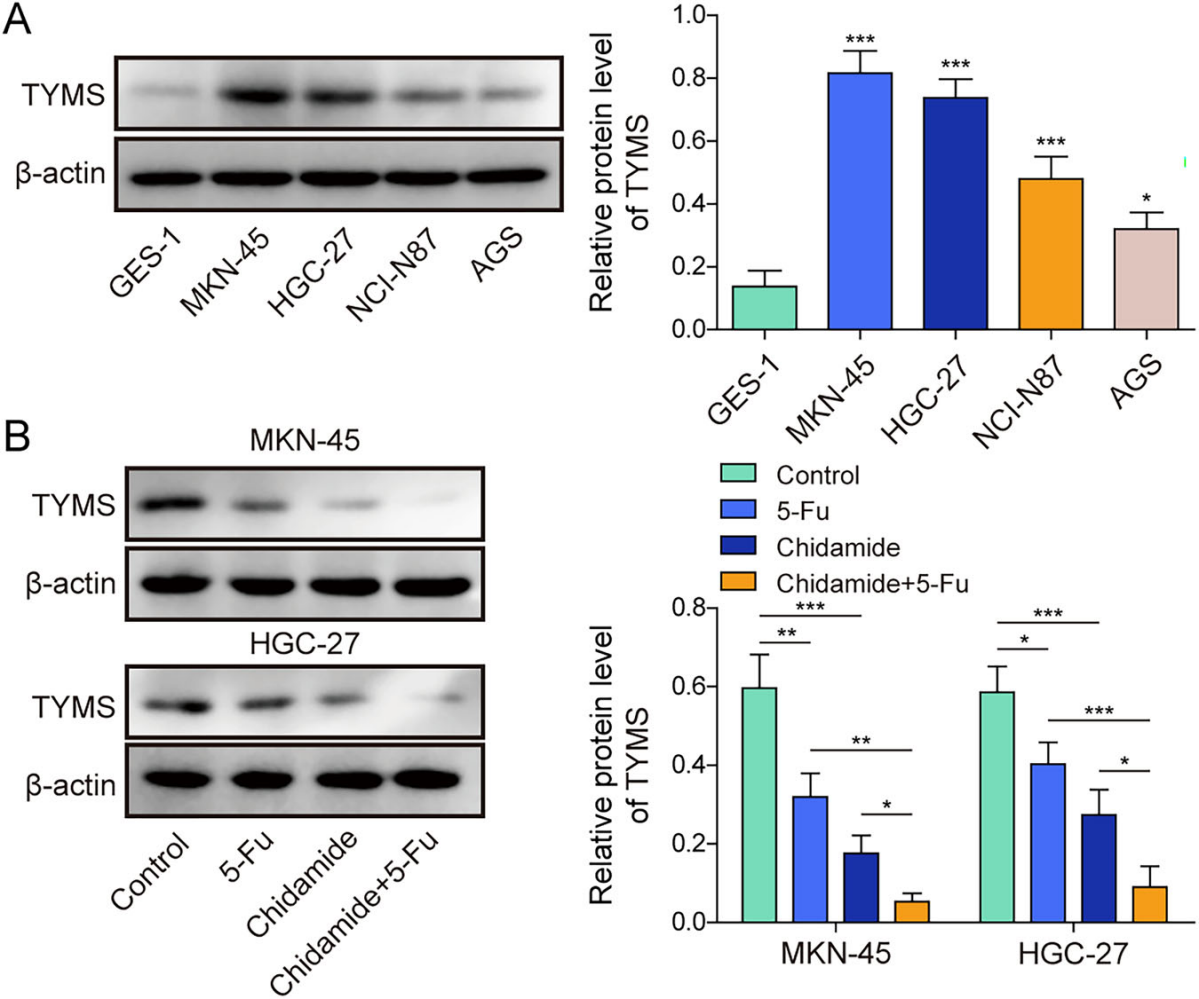
Chidamide enhances the sensitivity of gastric cancer cells to 5-FU. **A** The expression of TYMS in gastric mucosal epithelial cells, GES-1, and gastric cancer cell lines, MKN-45, HGC-27, NCI-N87, and AGS, was assessed by Western blot. **B** GC cell lines, including MKN-45, HGC-27, were exposed to 5 µg/mL of 5-FU and/or chidamide at 30 µM for 48 h. The expression of TYMS was measured by Western blot. All experiments were repeated at least 3 times, *p < 0.05, **p < 0.01, ***p < 0.001.

To determine the effective concentrations of chidamide and 5-FU for treatment, GC cell lines MKN-45, HGC-27, and NCI-N87 were treated with various concentrations of these agents. Chidamide exhibited no effect on cell viability at concentrations below 1 µM but demonstrated an inhibitory effect above this threshold in a concentration-dependent manner in MKN-45 and HGC-27 cells, while it remained ineffective against NCI-N87 cells (Fig. S1A). As viability results at 72 h were not significantly different from those at 48 h, subsequent experiments were limited to the 24- and 48-h time points to improve experimental efficiency. To further assess the baseline sensitivity of these GC cell lines to 5-FU, we evaluated their IC₅₀ values at 24-, 48-, and 72-h post-treatment. All three cell lines showed a time-dependent decrease in IC₅₀ values (Fig. S1B). Among them, MKN-45 consistently displayed the highest sensitivity to 5-FU, followed by HGC-27, while NCI-N87 showed the greatest resistance across all time points. These results supported the selection of MKN-45 and HGC-27 for subsequent mechanistic studies. Additionally, MKN-45, HGC-27, and NCI-N87 cell viability decreased in a concentration- and time-dependent manner when exposed to 5 µg/mL or more of 5-FU (Fig. S1B). Based on this observation, further experiments were conducted using 5 µg/mL of 5-FU for 48 h. When cells were treated with 10 µM of chidamide in conjunction with 5-FU, cell viability decreased by an additional 20%; at 30 µM of chidamide, the decrease was approximately 40% (Fig. S1C, D). For subsequent experiments, the combined treatment conditions for chidamide will be selected as 30 µM for 48 h. Quantitative analysis of drug interaction was performed using zero interaction potency (ZIP) model to calculate the ZIP synergy score. In MKN-45, HGC-27, and NCI-N87 cells, the ZIP values for chidamide and 5-FU co-treatment were between −10 and 10 at both 24 and 48 h (Fig. S2), indicating the presence of an additive effect. To investigate the molecular basis underlying chidamide-enhanced 5-FU sensitivity via HDAC3, we first analyzed the expression of 13 genes—DHFR, DPYS, DTYMK, DUT, FPGS, GGH, NME1, NT5C, RRM1, TYMS, UCK2, UNG, and UMPS—known to regulate 5-FU pharmacodynamics and cellular chemosensitivity. As shown in Fig. S3A, treatment with chidamide led to reduced mRNA expression of DPYS, TYMS, and UNG. Chidamide inhibited HDAC3 levels, whereas 5-FU did not affect HDAC1/2/3/10 in all GC cells (NCI-N87, MKN-45 and HGC-27) (Fig. S3B). Besides, the combined treatment further reduced HDAC3 levels (Fig. S3B). Using ChIPBase (https://rnasysu.com/chipbase3/index.php), we predicted HDAC3 binding sites within the promoter regions of the 13 genes mentioned above. Notably, HDAC3 binding was predicted only at the promoters of *FPGS*, *NME1*, *NT5C*, and *TYMS* (Fig. S3C). Based on this overlap, TYMS was identified as the most relevant downstream target via Venn diagram analysis (Fig. S3D). Besides, only TYMS expression was reduced following HDAC3 knockdown (Fig. S3E). Survival analysis revealed that high expression of TYMS was significantly associated with poor overall survival in GC patients (Fig. S4A). As shown in Fig. 1A, TYMS expression was significantly higher in GC cells, including MKN-45, HGC-27, NCI-N87, and AGS, compared to normal gastric mucosal epithelial cells GES-1. TYMS expression was significantly higher in GC cells, including MKN-45, HGC-27, NCI-N87, and AGS, compared to normal gastric mucosal epithelial cells GES-1 (Fig. 1A). Among these, MKN-45, and HGC-27, showing the highest elevated TYMS levels, were chosen for deeper investigations. Both 5-FU and chidamide independently reduced TYMS expression, with their combination leading to a greater reduction (Fig. 1B). These data suggest that chidamide enhances the sensitivity of GC cells to 5-FU.

### Chidamide promotes the sensitivity of gastric cancer cells to 5-FU by downregulating TYMS

**Fig. 2:**
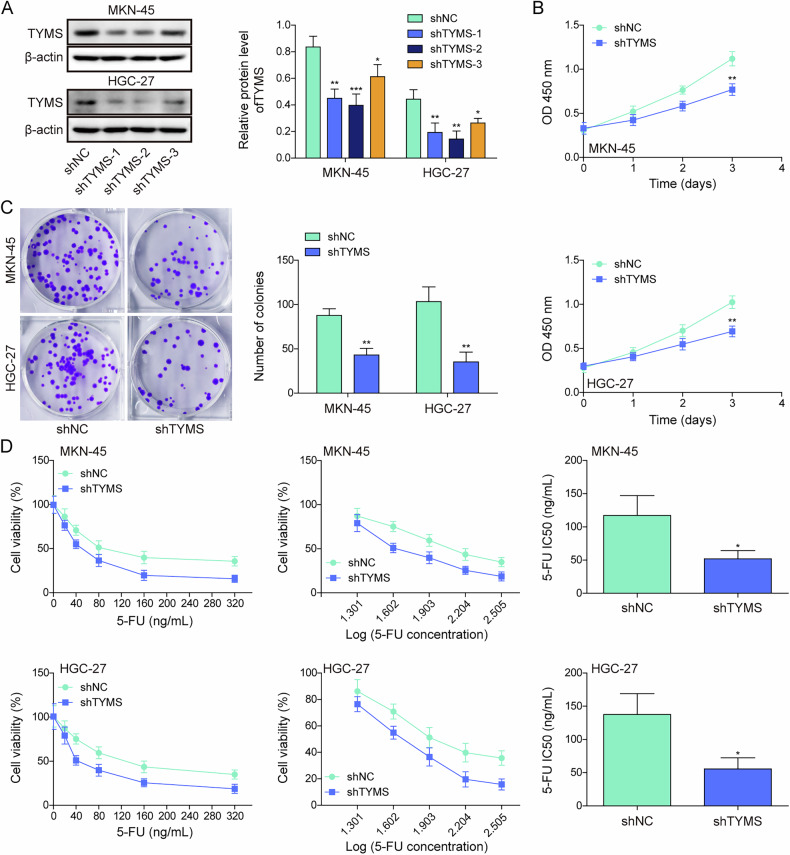
Chidamide promotes the sensitivity of gastric cancer cells to 5-FU by downregulating TYMS. **A** GC cell lines, including MKN-45, HGC-27, were transfected with shNC, shTYMS-1, shTYMS-2, and shTYMS-3, to construct TYMS knockdown cell lines. TYMS expression was analyzed using Western blot. **B**–**D** GC cell lines, including MKN-45, HGC-27, were transfected with shNC or shTYMS-1. **B** Cell viability was measured using the CCK-8 assay. **C** Cell proliferation was assessed via colony formation assay. **D** Sensitivity to 5-FU in GC cells was evaluated using the CCK-8 assay. All experiments were repeated at least three times, *p < 0.05, **p < 0.01, ***p < 0.001.

To explore the regulatory role of TYMS in GC, targeted knockdown of TYMS was performed in GC cell lines HGC-27 and MKN-45 using shRNA vectors sh-TYMS-1, sh-TYMS-2, and sh-TYMS-3. The knockdown efficiency was validated by Western blot, with sh-TYMS-2 achieving the highest knockdown efficiency and selected for subsequent analyses (Fig. 2A). TYMS silencing markedly decreased cell viability, proliferation, and enhanced sensitivity to 5-FU in HGC-27 and MKN-45 cells (Fig. 2B–D). Subsequently, TYMS was overexpressed in these cell lines, resulting in a marked increase in TYMS protein levels (Fig. S5A). While chidamide treatment alone reduced TYMS expression, this suppressive effect was partially reversed in cells with enforced TYMS overexpression (Fig. S5A). Increased TYMS expression enhanced cell viability and proliferation while reducing sensitivity to 5-Fu (Fig. S5B–D). Conversely, chidamide addition yielded opposite outcomes. Furthermore, TYMS overexpression was able to counteract the effects of chidamide on cell viability, proliferation, and the enhanced sensitivity of HGC-27 and MKN-45 cells to 5-FU. Taken together, chidamide treatment increases the responsiveness of GC cells to 5-FU through the downregulation of TYMS.

### Chidamide relieved the suppressive effect of HDAC3 on the sensitivity of gastric cancer cells to 5-FU

**Fig. 3:**
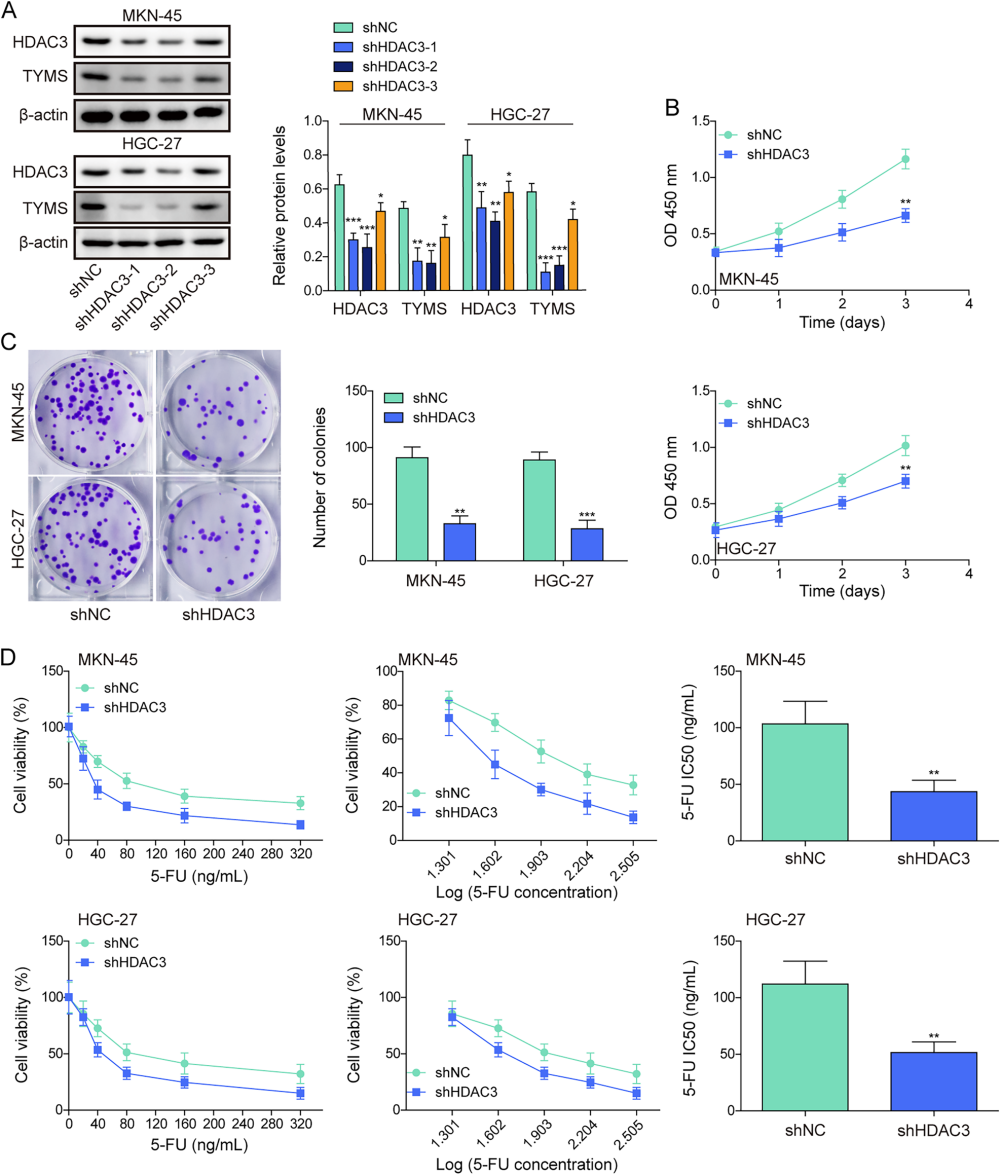
Chidamide relieved the suppressive effect of HDAC3 on the sensitivity of gastric cancer cells to 5-FU. **A** GC cell lines, including MKN-45, HGC-27, were transfected with shNC, shHDAC3-1, shHDAC3-2, and shHDAC3-3, to construct HDAC3 knockdown cell lines. HDAC3 and TYMS expression was analyzed using Western blot. **B**–**D** GC cell lines, including MKN-45, HGC-27, were transfected with shNC or shHDAC3-2. **B** Cell viability was measured using the CCK-8 assay. **C** Cell proliferation was assessed via colony formation assay. **D** Sensitivity to 5-FU in GC cells was evaluated using the CCK-8 assay. All experiments were repeated at least three times, *p < 0.05, **p < 0.01, ***p < 0.001.

To elucidate the role of the HDAC family in modulating the sensitivity of GC cells to 5-FU, treatments with chidamide alone or combined with 5-FU were administered. As presented in Fig. S3B, chidamide inhibited HDAC3 levels, and the combined treatment further reduced HDAC3 levels (Fig. S3B). It was also revealed by survival analysis that high expression of HDAC3 was significantly correlated with poor overall survival in GC patients (Fig. S4B). Subsequently, HDAC3 was knocked down in MKN-45 and HGC-27 to further investigate its role. Knockdown of HDAC3 resulted in decreased expression of HDAC3 and TYMS, with shHDAC3-2 showing the most significant effect and was chosen for ongoing studies (Fig. 3A). HDAC3 depletion markedly decreased cell viability, proliferation, and enhanced sensitivity to 5-FU (Fig. 3B–D). Overexpression of HDAC3 in these cells increased HDAC3 and TYMS levels, while chidamide showed the opposite effect (Fig. S6A). Notably, HDAC3 overexpression could reverse the inhibitory effects of chidamide on HDAC3 and TYMS expression. Moreover, overexpression of HDAC3 enhanced cell viability and proliferation while reducing sensitivity to 5-FU (Fig. S6B–D). Additionally, overexpression of HDAC3 effectively counteracted the inhibitory effect of chidamide on cell viability, proliferation and 5-FU sensitivity. Altogether, HDAC3 represses the sensitivity of GC cells to 5-FU; however, this effect is relieved following treatment with chidamide.

### The HDAC3/HNF4A complex regulates the transcriptional activity of *TYMS*

**Fig. 4:**
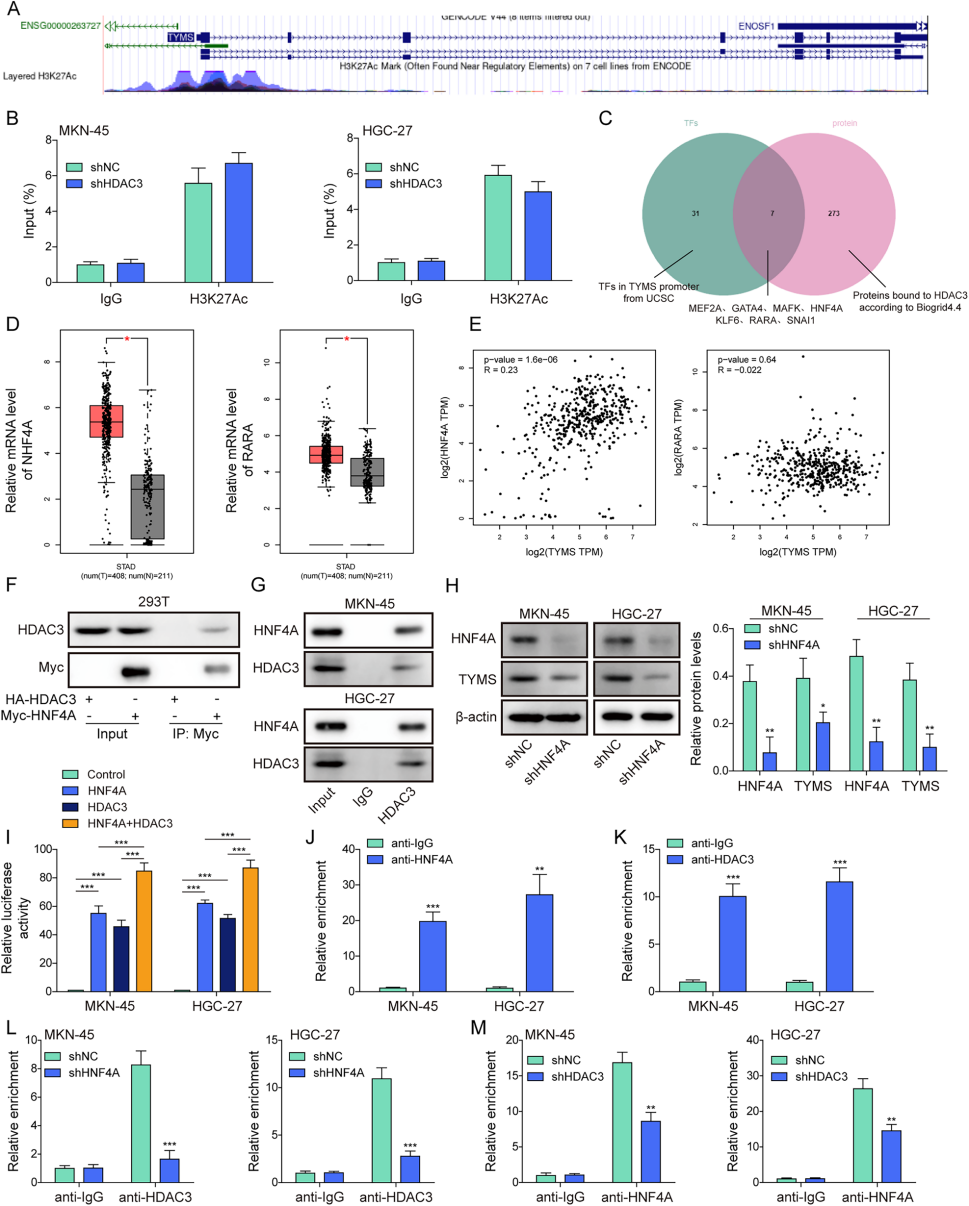
The HDAC3/HNF4A complex regulates the transcriptional activity of *TYMS*. **A** Information on H3K27Ac enrichment on the *TYMS* promoter region was acquired from the UCSC website. **B** GC cell lines, including MKN-45, HGC-27, were transfected with shNC or shHDAC3-2. H3K27Ac enrichment on the *TYMS* promoter was detected using ChIP. **C** Transcription factors (TFs) associated with the *TYMS* promoter were selected utilizing the UCSC website (threshold set at Minimum Score: 500), and TFs binding with HDAC3 were screened using the BioGRID4.4. An intersection was identified including HNF4A, MEF2A, GATA4, MAFK, KLF6, RARA, SNAI1. **D** The GEPIA database was used to screen the TFs, and the upregulated TFs (RARA and HNF4A) were presented. **E** The correction between HNF4A, RARA and TYMS were predicted based on the GEPIA database. **F** The binding of exogenous HDAC3 and HNF4A was tested using Co-IP in 293 T cells. **G** The binding of endogenous HDAC3 and HNF4A was detected in MKN-45 and HGC-27 cells using Co-IP. **H** The expression of HNF4A and TYMS in HNF4A depleted MKN-45 and HGC-27 cells was analyzed using Western blot. **I** Luciferase activity on the *TYMS* promoter was evaluated in HDAC3 and/or HNF4A overexpressed MKN-45 and HGC-27 cells using a dual-luciferase reporter assay. **J** The binding of HNF4A to the *TYMS* promoter was analyzed in MKN-45 and HGC-27 cells using ChIP-qPCR. **K** The binding of HDAC3 to the *TYMS* promoter was analyzed in MKN-45 and HGC-27 cells using ChIP-qPCR. **L** The binding of HDAC3 to the *TYMS* promoter in HNF4A knockdown MKN-45 and HGC-27 cells was tested using ChIP-qPCR. **M** The binding of HNF4A to the *TYMS* promoter in HDAC3-depleted MKN-45 and HGC-27 cells was detected using ChIP-qPCR. All experiments were repeated at least three times, *p < 0.05, **p < 0.01, ***p < 0.001.

To further explore how chidamide regulates TYMS expression and the regulatory role of HDAC3 in this mechanism, we analyzed the promoter region of *TYMS* using the online bioinformatic tool. Chidamide mainly inhibited H3K27Ac, but not H3K9Ac, H3K18Ac, H3K14Ac, and H4K8Ac (Fig. S4C). The UCSC indicated H3K27Ac enrichment at the *TYMS* promoter (Fig. 4A). Subsequent ChIP assays confirmed that this enrichment remained unchanged following HDAC3 knockdown (Fig. 4B). Screening for transcription factors associated with the *TYMS* promoter through the UCSC website, further refined by the BioGRID4.4 website for interactions with HDAC3, identified seven transcription factors (TFs): HNF4A, MEF2A, GATA4, MAFK, KLF6, RARA, and SNAI1 (Fig. 4C). The GEPIA database was then utilized to further assess these TFs, highlighting HNF4A and RARA as highly expressed in stomach cancer (STAD), with subsequent analyses indicating a significant positive correlation between HNF4A and TYMS (Fig. 4D, E). RARA, however, showed no significant correlation, prompting the selection of HNF4A for further study. It was indicated that HNF4A high expression was closely associated with poorer patient outcomes (Fig. S4D). Exogenous interaction between HDAC3 and HNF4A was confirmed by Co-IP in 293T cells (Fig. 4F), with endogenous binding verified in GC cells, HGC-27 and MKN45 (Fig. 4G). Knockdown of HNF4A in HGC-27 and MKN45 cells decreased the expression of both HNF4A and TYMS (Fig. 4H). Overexpression of HDAC3 and/or HNF4A in 293T cells led to an increase in *TYMS* promoter activity (Fig. 4I**)**. ChIP-qPCR experiments in GC cells further validated the binding of HNF4A and HDAC3 to the *TYMS* promoter (Fig. 4J, K). Additionally, the knockdown of HNF4A almost entirely reduced HDAC3 binding to the *TYMS* promoter (Fig. 4L), while the knockdown of HDAC3 partly decreased the binding of HNF4A to the *TYMS* promoter (Fig. 4M). This suggests that HNF4A serves a critical regulatory function as a transcription factor, while HDAC3 acts as a transcriptional co-factor, impacting HNF4A and thus playing a role in transcription regulation.

### Phosphorylation of HNF4A at S313 promotes the expression of TYMS

**Fig. 5:**
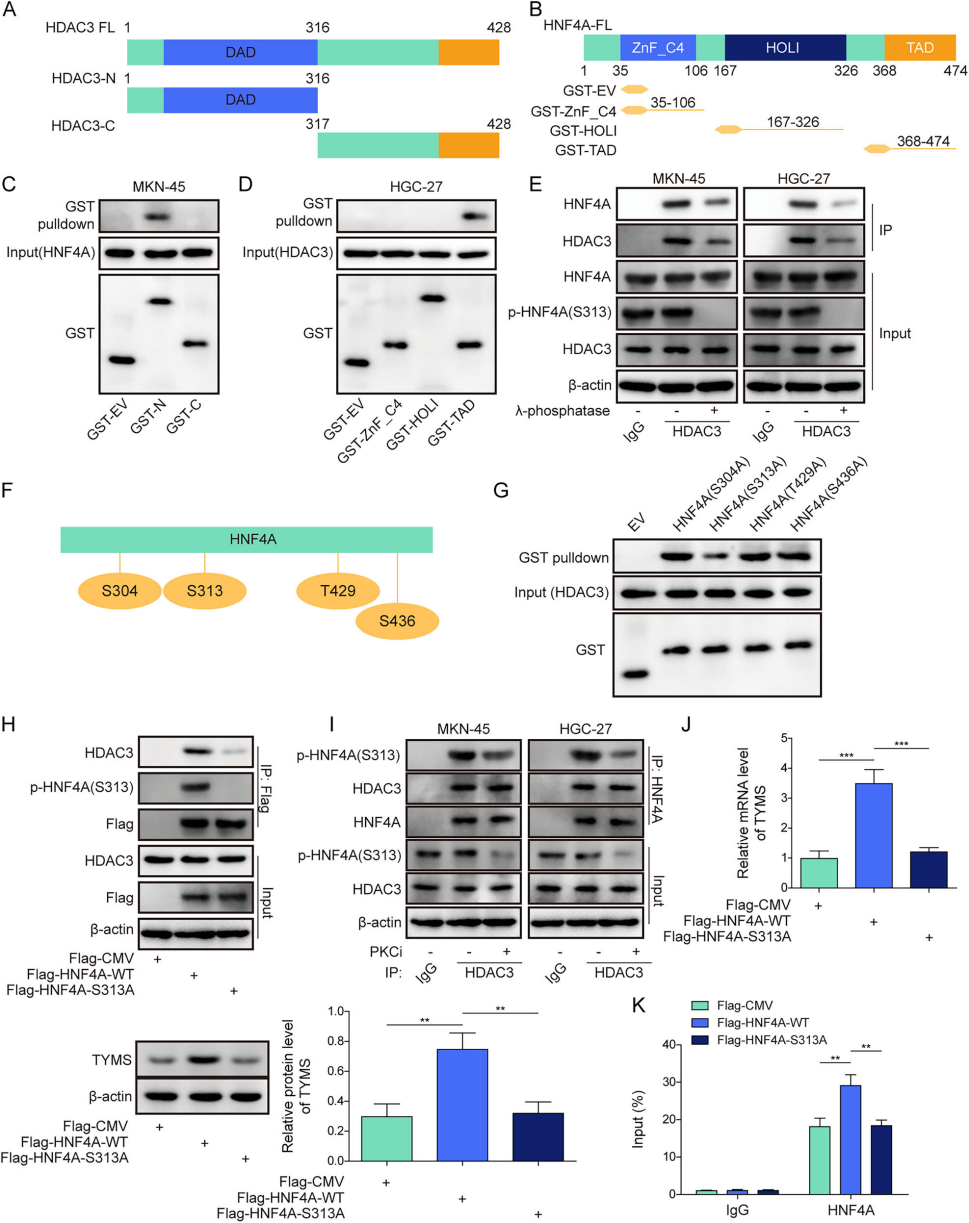
Phosphorylation of HNF4A at S313 promotes the expression of TYMS. 293T cells were used to construct various truncations of **A** HDAC3 and **B** HNF4A. **C** The interaction between these HDAC3 truncations and HNF4A was analyzed using GST-pulldown. **D** The interaction between these HNF4A truncations and HDAC3 was examined using GST-pulldown. **E** MKN-45 and HGC-27 cells were treated with λ-phosphatase. HNF4A phosphorylation was assessed using Western blot. In addition, the binding between HDAC3 and HNF4A was investigated using Co-IP. **F** A schematic of HNF4A phosphorylation sites is illustrated. **G** 293T cells were used to construct mutants of HNF4A at phosphorylation sites, including HNF4A-S304A, HNF4A-S313A, HNF4A-S436A and HNF4A-T429A. The interaction between HDAC3 and HNF4A was assessed using GST-pulldown to screen phosphorylation sites. **H** 293T cells were used to construct a mutant of HNF4A at the phosphorylation site, including HNF4A-S313A. The binding between HDAC3 and HNF4A was investigated using Co-IP. **I** MKN-45 and HGC-27 cells were treated with PKCi. The interaction between HDAC3 and HNF4A was examined using Co-IP. 293T cells were used to construct a mutant of HNF4A at the phosphorylation site, including HNF4A-S313A. **J** The expression of TYMS was evaluated using RT-qPCR and Western blot. **K** The binding of HNF4A to the TYMS promoter was analyzed using ChIP. All experiments were repeated at least 3 times, *p < 0.05, **p < 0.01, ***p < 0.001.

To elucidate the mechanism by which the HDAC3/HNF4A complex regulates TYMS expression, various truncations of HDAC3 and HNF4A were constructed (Fig. 5A, B). GST-pulldown assays revealed that HNF4A binds to HDAC3’s N-terminus containing the deacetylase-activating domain (DAD), and HDAC3 binds to the transactivation domain (TAD) of HNF4A (Fig. 5C, D). To further confirm this interaction, we examined whether the HDAC3 DAD domain alone is sufficient for binding HNF4A. As shown in Fig. S7A, GST-tagged HDAC3-DAD efficiently pulled down HNF4A. Treatment with λ-phosphatase decreased phosphorylation of HNF4A in HGC-27 and MKN45 cells, diminishing its interaction with HDAC3 (Fig. 5E). To better understand which post-translational modifications of HNF4A are functionally relevant in the context of chidamide treatment, we examined publicly available PTM databases, including PhosphoSitePlus, UniProt, and GeneCards, which indicated that HNF4A may undergo phosphorylation, acetylation, and O-glycosylation. We then treated MKN45 and HGC-27 cells with chidamide and assessed changes in these PTMs. The results demonstrated a marked increase in acetylation, a decrease in phosphorylation, and no change in O-glycosylation (Fig. S7B). These findings led us to focus on phosphorylation as one of the key regulatory mechanisms. Phosphorylation site predictions using NetPhos-3.1 identified T429 and S436 as highly credible sites, with S304 and S313 previously reported in the literature [22] (Fig. 5F). Mutations at these known (S304/S313) and predicted (T429/S436) sites showed that a mutation at S313 significantly reduced the binding between HDAC3 and HNF4A (Fig. 5G). This reduction was further validated by Co-IP, underscoring the critical impact of the S313A mutation on their interaction (Fig. 5H). The addition of PKCi resulted in a decrease in HNF4A phosphorylation, correlating with reduced binding to HDAC3, as assessed by Co-IP (Fig. 5I). Transfection of wild-type (WT)-HNF4A significantly increased TYMS mRNA and protein levels, as well as enhanced luciferase activity at the *TYMS* promoter. In contrast, cells transfected with the HNF4A S313A mutation exhibited no changes in TYMS mRNA or protein levels, nor promoter luciferase activity (Fig. 5J, K). Collectively, the expression of TYMS was increased by phosphorylation of HNF4A.

### HDAC3 deacetylates HNF4A at K458, while chidamide enhances HNF4A acetylation at K458 and reduces phosphorylation at S313

**Fig. 6:**
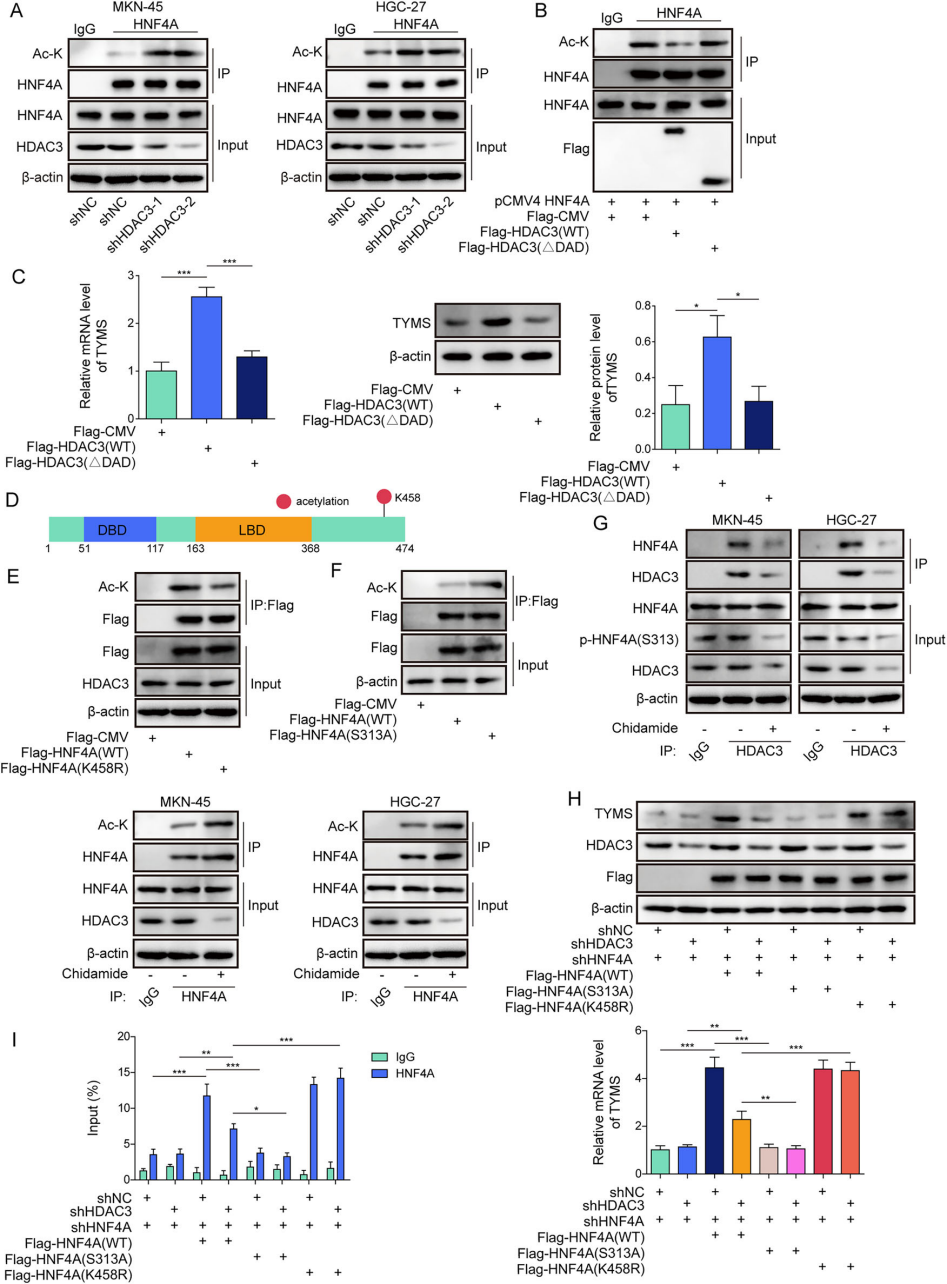
HDAC3 deacetylates HNF4A at K458, while chidamide enhances HNF4A acetylation at K458 and reduces phosphorylation at S313. **A** Co-IP was used to analyze the acetylation modification of HNF4A by HDAC3 in HDAC3 depleted MKN-45 and HGC-27 cells. **B**, **C** 293T cells were utilized to construct various truncations of HDAC3, including wide type HDAC3 or DAD-deleted HDAC3. **B** Afterward, Co-IP was used to assess the acetylation modification of HNF4A by HDAC3. **C** The expression of TYMS was evaluated using RT-qPCR and Western blot in 293T cells. **D** A schematic of HNF4A acetylation sites is presented. **E** 293T cells were used to construct mutants of HNF4A at acetylation sites, including HNF4A-K458R. Co-IP was employed to analyze the acetylation status of HNF4A. **F** 293T cells were used to construct mutants of HNF4A at phosphorylation sites, such as HNF4A-S313A. Co-IP was used to analyze the acetylation status of HNF4A. **G** MKN-45 and HGC-27 cells. were treated with chidamide. Co-IP was employed to examine the interaction between HDAC3 and HNF4A, and the acetylation status of HNF4A. **H**–**I** 293T cells were used to construct mutants of HNF4A at acetylation sites, with concurrent knockdown of HDAC3 or/and HNF4A. **H** TYMS expression was analyzed using RT-qPCR, and the expression of Flag, HDAC3, and TYMS was assessed using Western blot. **I** ChIP was used to detect the binding of HNF4A to the TYMS promoter. All experiments were repeated at least three times, *p < 0.05, **p < 0.01, ***p < 0.001.

Next, we investigated whether and how HDAC3 and chidamide regulate the acetylation and phosphorylation of HNF4A. Knocking down HDAC3 led to an increase in the acetylation levels of HNF4A (Fig. 6A). Conversely, WT-HDAC3 transfection decreased HNF4A’s acetylation levels and increased TYMS expression. Deleting the DAD in HDAC3 increased HNF4A acetylation and decreased TYMS expression (Fig. 6B, C). The K458 site was identified as acetylate on HNF4A (Fig. 6D). Compared with the transfection of WT-HNF4A, the HNF4A-K458R mutation led to reduction in acetylation (Fig. 6E). Additionally, the transfection of HNF4A-S313A mutation increased HNF4A’s acetylation, suggesting that inhibited phosphorylation affected acetylation levels (Fig. 6F).

To further clarify the crosstalk between these two modifications, we transfected MKN-45 cells with either WT-HNF4A or the K458R mutant. Co-IP assays revealed that the K458R mutation reduced the interaction between HNF4A and HDAC3, and simultaneously increased S313 phosphorylation, indicating that acetylation at K458 negatively regulates phosphorylation at S313 (Fig. S8A). Furthermore, treatment with the HDAC3-specific inhibitor RGFP966 reduced the HNF4A-HDAC3 interaction and reduced S313 phosphorylation, confirming that HDAC3 modulates HNF4A phosphorylation indirectly through deacetylation (Fig. S8B). Treatment with chidamide reduced the phosphorylation levels of HNF4A, decreased the interaction between HDAC3 and HNF4A, and increased acetylation of HNF4A (Fig. 6G). To further explore the upstream regulation of HNF4A phosphorylation, MKN-45 cells were transfected with Flag-HDAC3 and treated with the AMPK inhibitor BAY-3827. Co-IP assays demonstrated that AMPK inhibition decreased S313 phosphorylation and decreased HDAC3-HNF4A binding, indicating that kinase signaling regulates the recruitment of HDAC3 through HNF4A phosphorylation (Fig. S8C).

Knockdown of HNF4A resulted in reduced TYMS mRNA and protein expression, and decreased promoter activity (Fig. 6H, I). In contrast, transfections with either WT-HNF4A or HNF4A-K458R mutation increased TYMS expression and promoter activity. However, knocking down HDAC3 and transfecting WT-HNF4A decreased both TYMS expression and promoter activity, whereas knocking down HDAC3 and transfecting HNF4A-K458R mutation did not affect TYMS expression or promoter activity. Moreover, transfection of the HNF4A-S313A mutation did not change TYMS expression or promoter activity (Fig. 6H, I). Altogether, HDAC3 functions to deacetylate HNF4A at K458, whereas chidamide enhances the acetylation of HNF4A at K458 and diminishes phosphorylation at S313.

To further elucidate the mechanism by which chidamide modulates TYMS expression, we examined whether its regulatory effect was primarily mediated through inhibiting HDAC3 and its downstream target gene expression or through HNF4A acetylation. First, we treated MKN45 and HGC-27 cells with chidamide and performed ChIP assays to assess H3K27Ac levels at the *TYMS* promoter (Fig. S9A). No significant changes in H3K27Ac enrichment were observed following chidamide treatment. Next, to evaluate the effect of chidamide on HNF4A acetylation, we conducted Co-IP assays in 293T cells transfected with either WT Flag-HNF4A or an acetylation-deficient mutant Flag-HNF4A(K458R), in the presence or absence of chidamide (Fig. S9B). Chidamide significantly increased the acetylation of WT HNF4A, but not the K458R mutant. These findings were further confirmed in MKN45 and HGC-27 cells (Fig. S9C). Collectively, while chidamide inhibits HDAC3 and its downstream targets, the enhancement of HNF4A acetylation plays a more critical role in regulating TYMS expression and mediating chidamide’s sensitizing effect to 5-FU in GC cells.

### Chidamide suppresses the HDAC3/HNF4A/TYMS axis, thereby increasing the sensitivity of gastric cancer cells to 5-FU

**Fig. 7:**
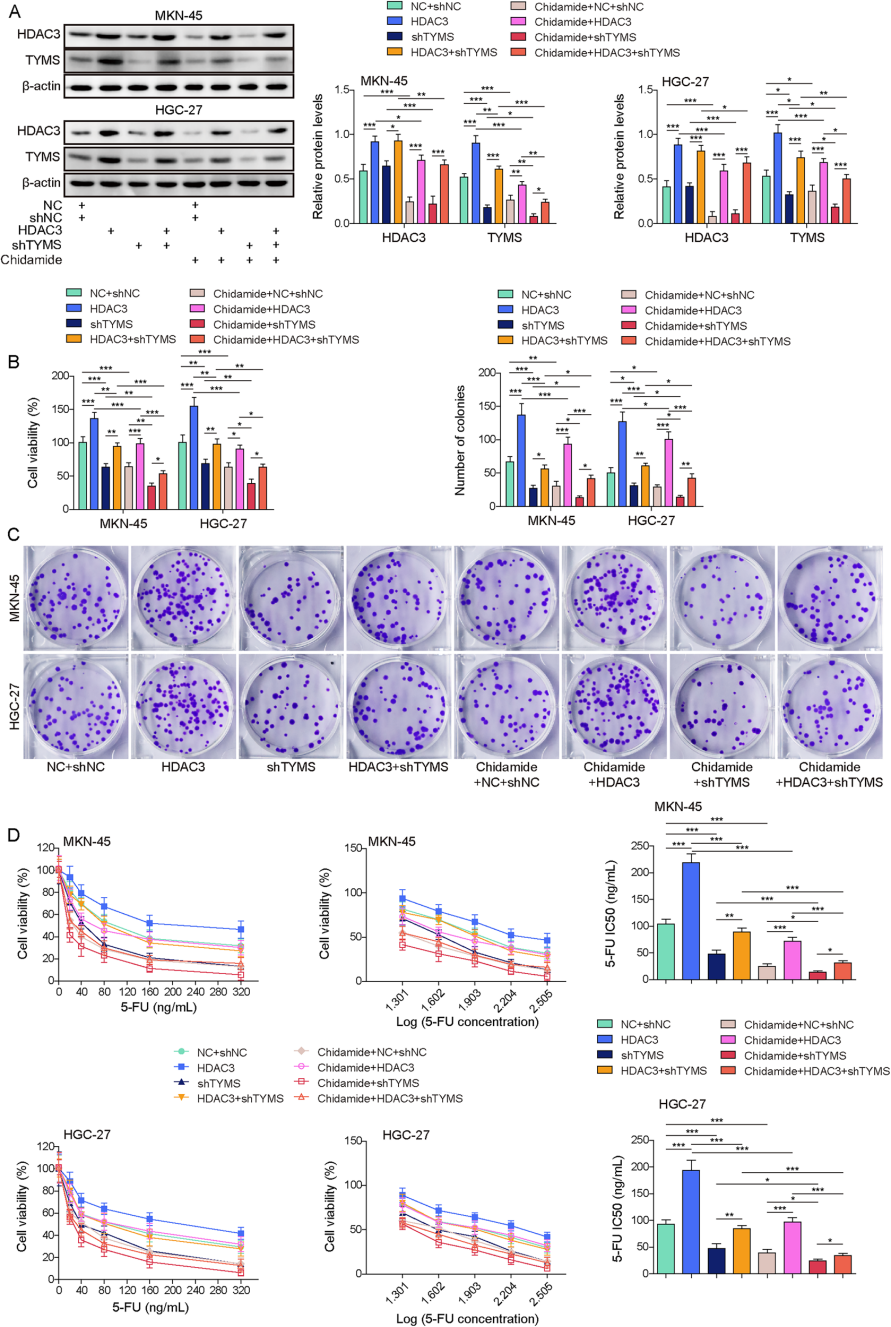
Chidamide suppresses the HDAC3/HNF4A/TYMS pathway, thereby increasing the sensitivity of gastric cancer cells to 5-FU. GC cell lines, including MKN-45, HGC-27, were transfected with shNC, shTYMS and/or OE-HDAC3. Afterward, the cells were treated with chidamide (30 µM). **A** TYMS and HDAC3 expression was analyzed using Western blot. **B** Cell viability was measured using the CCK-8 assay. **C** Cell proliferation was assessed via colony formation assay. **D** Sensitivity to 5-FU in GC cells was evaluated using the CCK-8 assay. All experiments were repeated at least 3 times, *p < 0.05, **p < 0.01, ***p < 0.001.

The role of the HDAC3/HNF4A/TYMS axis in the chidamide-modulated sensitivity of GC cells to drugs was further elucidated by knocking down TYMS while overexpressing HDAC3 in GC cells following chidamide treatment. Knocking down TYMS reversed the increase of TYMS expression induced by HDAC3 overexpression in HGC-27 and MKN45 cells (Fig. 7A). Additionally, the addition of chidamide led to a reduction in the expression levels of both HDAC3 and TYMS. Regarding cell behavior, knocking down TYMS reversed the promotional effects of HDAC3 overexpression on HGC-27 and MKN45 cell viability, proliferation, and resistance to 5-FU (Fig. 7B–D). Chidamide also contributed to reductions in cell viability, proliferation, and 5-FU resistance. Taken together, chidamide enhances the sensitivity of GC cells to 5-FU by inhibiting the HDAC3/HNF4A/TYMS expression.

### Chidamide promotes the sensitivity of GC to 5-FU by inhibiting HDAC3/HNF4A/TYMS axis in vivo

**Fig. 8:**
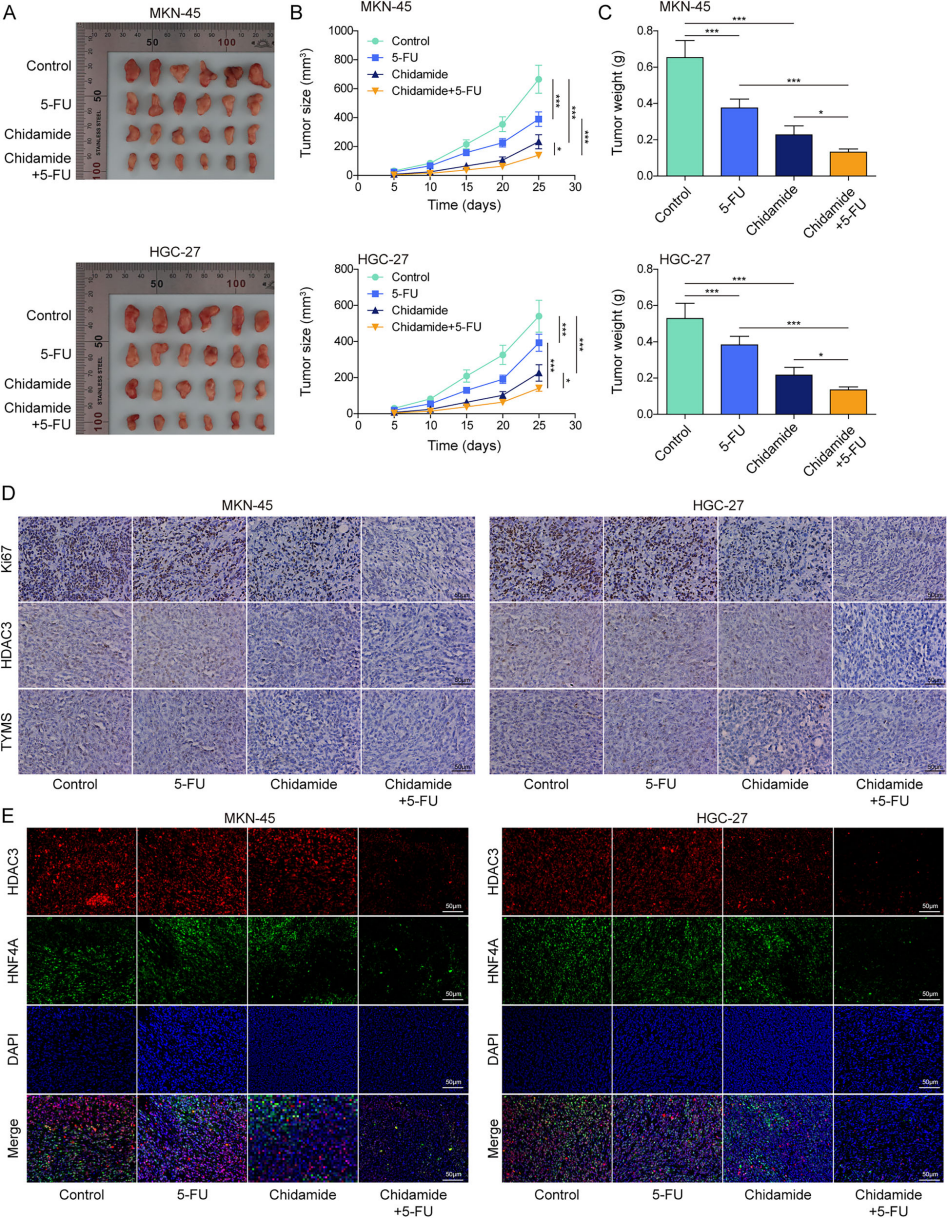
Chidamide promotes the sensitivity of GC to 5-FU by inhibiting HDAC3/HNF4A/TYMS axis in vivo*.* The GC mouse model was established, and the mice were treated with 5-FU and/or chidamide. The images of the tumors were shown in **A**. The tumor (**B**) volume and (**C**) weight were calculated. **D** The expression levels of Ki67, HDAC3, and TYMS were measured using IHC staining. Scale bar = 50 µm. **E** The colocalization of HDAC3 and HNF4A were assessed using IF staining. Scale bar = 50 µm. *N* = 6, *p < 0.05, ***p < 0.001.

Ultimately, the in vitro findings were corroborated using an in vivo mouse model. HGC-27 cells or MKN-45 cells were subcutaneously injected into the right dorsal midline of mice. Subsequently, treatments were administered to the mice, consisting of PBS only, 5-FU only, chidamide only, and a combination of 5-FU and chidamide. The administration of 5-FU or chidamide resulted in a substantial reduction in tumor size, volume, and weight, with chidamide showing more significant effects (Fig. 8A–C). The combined treatment of 5-FU and chidamide exhibited a synergistic effect on tumor reduction. Treatment with 5-FU led to decreased expression levels of Ki67 and TYMS, whereas the expression levels of HDAC3 remained stable (Fig. 8D). On the other hand, chidamide treatment alone significantly decreased the expression levels of Ki67, HDAC3 and TYMS. The combination of chidamide with 5-FU further amplified the reduction in these markers. Additionally, while the co-localization of HDAC3 and HNF4A in the nucleus was not affected by 5-FU alone, chidamide treatment reduced their nuclear co-localization, and the effect was enhanced when chidamide was used in conjunction with 5-FU (Fig. 8E). In summary, chidamide enhances the sensitivity of GC to 5-FU through inhibiting HDAC3/HNF4A/TYMS axis in vivo.

## Discussion

For several decades, 5-FU has been a cornerstone in chemotherapy for GC [23]. Despite its longstanding use, resistance to 5-FU frequently develops in nearly all cases of GC, often within a median time to progression of 6 months [24]. Understanding the mechanisms behind this acquired resistance remains a critical area of research. In this study, we have shown that chidamide enhances the sensitivity of GC cells to 5-FU by downregulating TYMS and HDAC3. We further demonstrated that chidamide treatment increased the acetylation of HNF4A at K458 by inhibiting HDAC3, which correlated with reduced phosphorylation of HNF4A at S313. Our findings suggest that chidamide downregulated the HDAC3/HNF4A/TYMS axis, thereby enhancing the response of GC cells to 5-FU. This study lays the groundwork for potential combined therapies using chidamide and 5-FU in treating GC.

Chidamide selectively binds to and inhibits HDAC1, 2, 3, and 10, proving effective as an anti-tumor agent in various cancers, including multiple myeloma, non-small cell lung cancer, and breast cancer [10, 25, 26]. Previous studies have underscored its role in overcoming drug resistance; for example, Yin et al. demonstrated that chidamide mitigated resistance to imatinib or nilotinib in chronic myeloid leukemia with the T315I mutation through modulation of the Akt-autophagy pathway [27]. Another recent study indicated its capacity to reverse fluzoparib resistance in triple-negative breast cancer cells [28]. Moreover, chidamide prompts BTG1-mediated autophagy, countering chemotherapy resistance in relapsed/refractory B-cell lymphoma [29]. HDAC3, one of chidamide’s targets, has been implicated in chemoresistance in various malignancies: it is essential for Kras-mutant lung cancer progression by co-regulating transcription with NKX2-1 and promoting FGFR1 expression, with its inhibition restoring trametinib sensitivity [30]. Similarly, in acute myeloid leukemia, HDAC3 promoted chemotherapy resistance by deacetylating and activating AKT in response to genotoxic stress [31]. Consistent with these findings, our study highlights chidamide’s role in enhancing GC cell sensitivity to 5-FU by reducing the expression of HDAC3 and TYMS. HNF4, a transcription factor in the nuclear receptor family, primarily functions in the liver and gastrointestinal tract and has been well-documented in the regulation of GC [32]. HNF4A has emerged as a targetable oncoprotein in GC, regulated by AMPK signaling via AMPKα [14]. Additionally, the HNF4A-BAP31-VDAC1 axis synchronously regulated cell ferroptosis and proliferation in GC [33]. A study even proposed HNF4A as a potential gold standard marker for distinguishing primary GC from breast metastasis [34]. Our study further confirmed the regulatory role of HNF4A in GC. We revealed a novel interaction between HNF4A and HDAC3, which forms a complex that transcriptionally modulates *TYMS* activity. Interestingly, prior research has indicated a correlation between HNF4A and HDAC3. Specifically, HDAC3 and PROX1 were found to co-occupy numerous genomic binding sites, showing a strong correlation with both the DNA-binding motif and the cistrome of HNF4A in mouse liver [35]. This observation further supports our study and underscores the potential significance of the interplay between HNF4A and HDAC3 in the context of GC regulation.

PTMs such as phosphorylation and acetylation play crucial roles in mediating the stability, localization, and DNA binding capacity of HNF4 [36]. For instance, phosphorylation at Ser78 (also known as Ser87), a conserved residue within the DNA-binding domain (DBD), has been identified to influence DNA binding, transactivation potential, protein stability, and nuclear localization adversely [37]. Furthermore, alterations in the acetylation pattern of HNF4A have been observed in human decompensated hepatocytes, notably in conditions like nonalcoholic steatohepatitis (NASH) and alcohol-induced Laennec’s cirrhosis in explanted livers [38]. Acetylation at K458 within the F domain significantly impacts HNF4A-mediated transcriptional control [39]. Notably, a mutant lacking acetylation at lys458 displayed approximately a twofold increase in transcriptional activity [39]. Furthermore, acetylation and phosphorylation often exhibit reciprocal regulation: acetylation can promote phosphorylation, as observed in the Tau protein at K280 [40, 41], or antagonize it [40–42], depending on the specific protein and modification site. The functional consequences of transcription factor phosphorylation can vary depending on the residue involved and the cellular environment, as exemplified by p38γ/δ-mediated modulation of MEF2D activity [43]. In our study, chidamide was found to promote HNF4A acetylation at K458 by disrupting its interaction with HDAC3. This modification coincided with reduced phosphorylation at S313 and downregulation of TYMS expression. Notably, mutation at K458 not only disrupted HDAC3 binding but also resulted in elevated S313 phosphorylation, suggesting coordinated regulation between these two PTMs. Our findings indicate that the increased acetylation of HNF4A, rather than a broad inhibition of HDAC3, plays a central role in repressing TYMS and enhancing 5-FU sensitivity. Moreover, the inhibition of AMPK decreased S313 phosphorylation and suppressed HDAC3-HNF4A binding, suggesting that upstream kinase signaling may influence HDAC3 recruitment through its phosphorylation status. These results provide strong evidence that chidamide regulates HNF4A modifications through inhibition of HDAC3 and reveal a novel mechanism by which chidamide suppresses TYMS expression and enhances 5-FU sensitivity in GC cells.

In conclusion, our findings elucidate that chidamide increases the sensitivity of GC to 5-FU by modulating the HDAC3/HNF4A/TYMS axis. These results suggest that chidamide treatment could be a promising strategy to combat GC resistance to 5-FU. While in vitro and in vivo mouse models offer valuable insights into the mechanisms of chidamide-mediated sensitivity to 5-FU, the complexity of the human physiological and immunological landscape may limit the direct applicability of these findings to clinical settings. Future research should aim to validate these results through more extensive preclinical and clinical studies. Moreover, potential off-target effects and hematologic toxicity associated with HDAC inhibitors, including chidamide, should be carefully considered in the context of combination therapy. Chidamide has been associated with adverse hematologic effects such as thrombocytopenia, neutropenia, and leukopenia [44]. These toxicities may limit its therapeutic window or necessitate dose optimization, patient selection, and close hematologic monitoring in future clinical applications. Future studies should aim to validate these results in more comprehensive preclinical models and clinical trials, with attention to both efficacy and safety profiles.

## Supplementary information

Supplementary Materials

## Data Availability

The raw data supporting the conclusions of this manuscript will be made available by the authors, without undue reservation, to any qualified researcher.
