# Supplementary material for: Chidamide enhances the sensitivity of gastric cancer to 5-fluorouracil chemotherapy by suppressing the HDAC3/HNF4A/TYMS axis
Source: Cell Death Dis. 2025 Dec 1;17(1):2. doi: 10.1038/s41419-025-08247-y (PMC12780113; doi:10.1038/s41419-025-08247-y)
Supplement: Supplementary file 1 — Supplementary Materials [file 41419_2025_8247_MOESM1_ESM.pdf]

Supplementary Materials

1. Supplementary Figure and Figure legends

Supplementary Figure 1. Cell viability for GC cell line under treatment with chidamide or 5-fluorouracil alone or in collaboration.

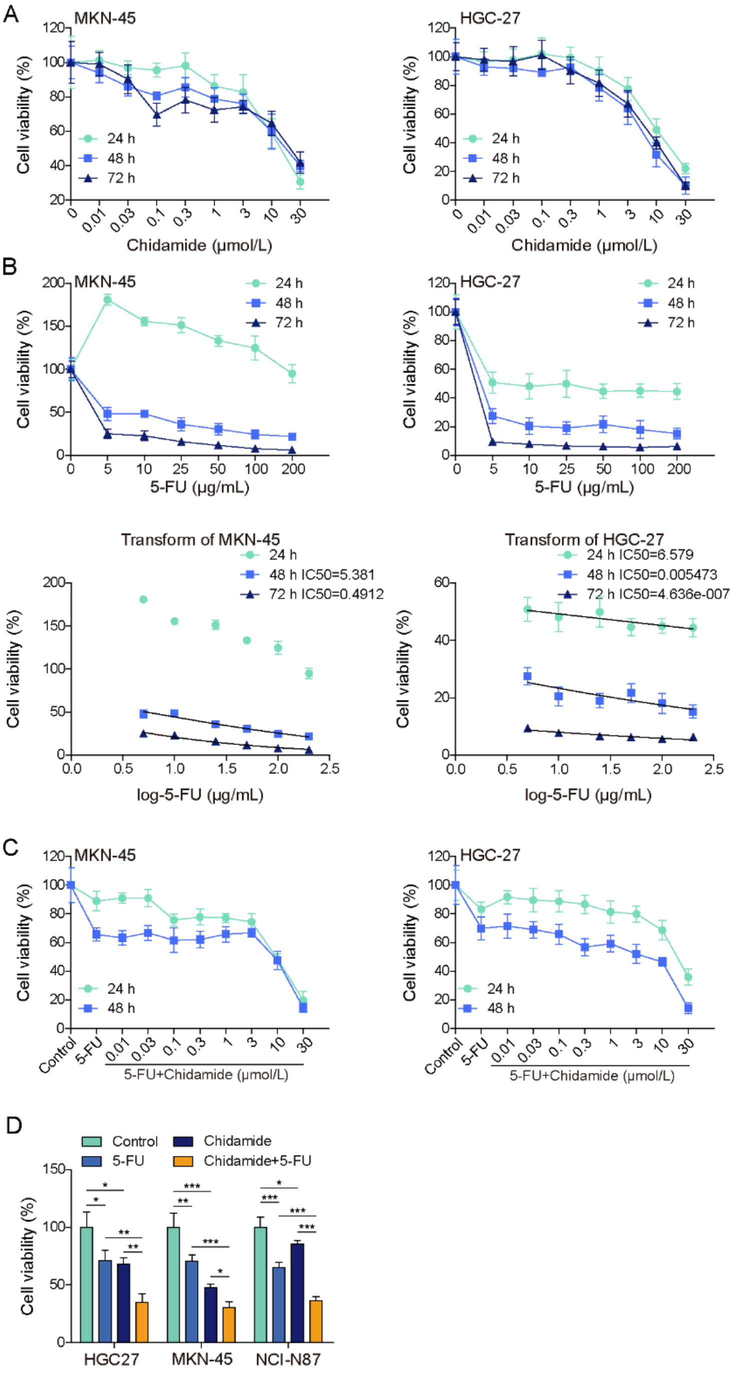

(A) GC cell lines, including MKN-45, HGC-27, NCI-N87, were treated with varied concentrations of chidamide (0, 0.01, 0.03, 0.1, 0.3, 1.0, 3.0, 10, 30 μmol/L) for 24, 48,

and 72 hours. Afterward, cell viability was assessed using the CCK-8 assay. (B) GC cell lines, including MKN-45, HGC-27, NCI-N87, were treated with 5-FU (0, 5, 10, 25, 50, 100, 200 μg/mL) for 24, 48, and 72 hours. Afterward, cell viability was measured using the CCK-8 assay, and IC_{50} values were calculated to assess time-dependent drug sensitivity. (C) GC cell lines, including MKN-45, HGC-27, NCI-N87, were treated for 48 hours with 5 μg/mL of 5-FU along with varying concentrations of chidamide (0, 0.01, 0.03, 0.1, 0.3, 1.0, 3.0, 10, 30 μmol/L). Cell viability was analyzed using the CCK-8 assay. (D) GC cell lines, including MKN-45, HGC-27, NCI-N87, were exposed to 5 μg/mL of 5-FU and chidamide at 30 μM for 48 hours. (D) Cell viability was evaluated using the CCK-8 assay. All experiments were repeated at least 3 times, *$p<0.05$, **$p<0.01$, ***$p<0.001$.

Supplementary Figure 2. Chidamide and 5-FU have an additive effect on each other.

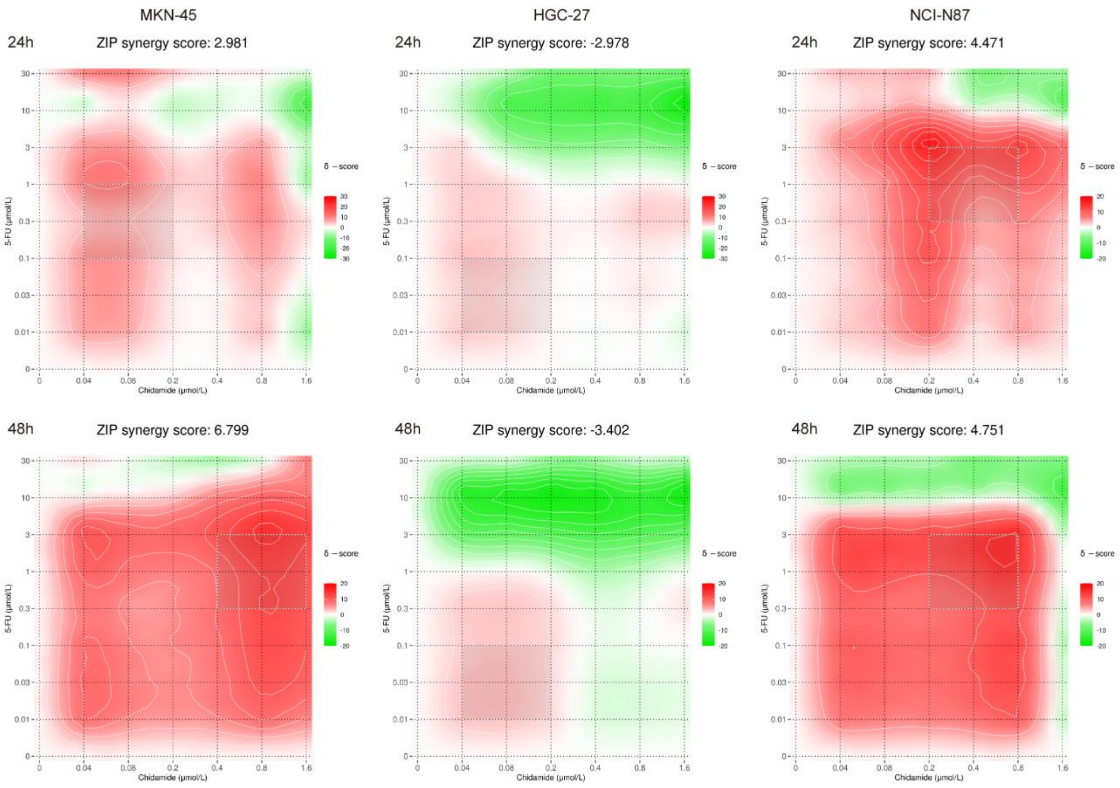

MKN-45, HGC-27, and NCI-N87 cells were treated with chidamide, 5-FU, or their combination for 24 and 48 hours. Zero interaction potency (ZIP) values for chidamide and 5-fluorouracil (5-FU) were calculated to assess drug interactions.

Supplementary Figure 3. Chidamide or suppressing HDAC3 downregulated TYMS.

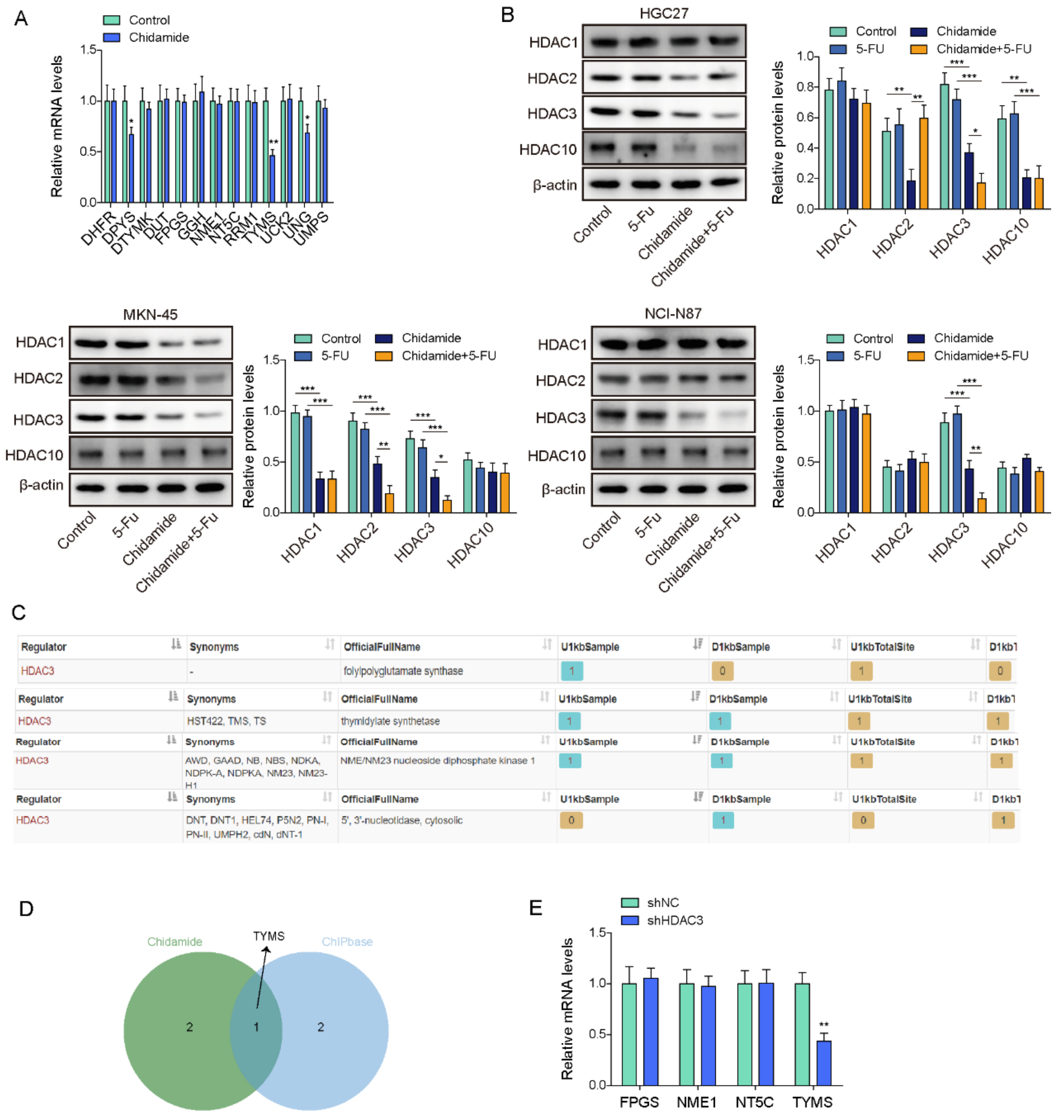

(A) RT-qPCR analysis of 13 genes involved in 5-FU pharmacodynamics in MKN-45 gastric cancer cells treated with chidamide. GC cell lines, including MKN-45, HGC-27, NCI-N87, were exposed to 5 µg/mL of 5-FU and chidamide at 30 µM for 48 hours. (B) HDAC1, HDAC2, HDAC3, and HDAC10 expressions were analyzed using Western blot. (C) ChIPBase-based prediction of HDAC3 binding sites within the promoter regions of the 13 genes. (D) Venn diagram showing the intersection between genes downregulated by chidamide and genes with predicted HDAC3 promoter binding. (E) The expression of FPGS, NME1, NT5C and TYMS was detected by RT-qPCR in

MKN-45 cells transfected with shNC and shHDAC3. All experiments were repeated at least 3 times, *p<0.05, **p<0.01, ***p<0.001.

Supplementary Figure 4. Survival analysis of TYMS, HDAC3 and HNF4A.

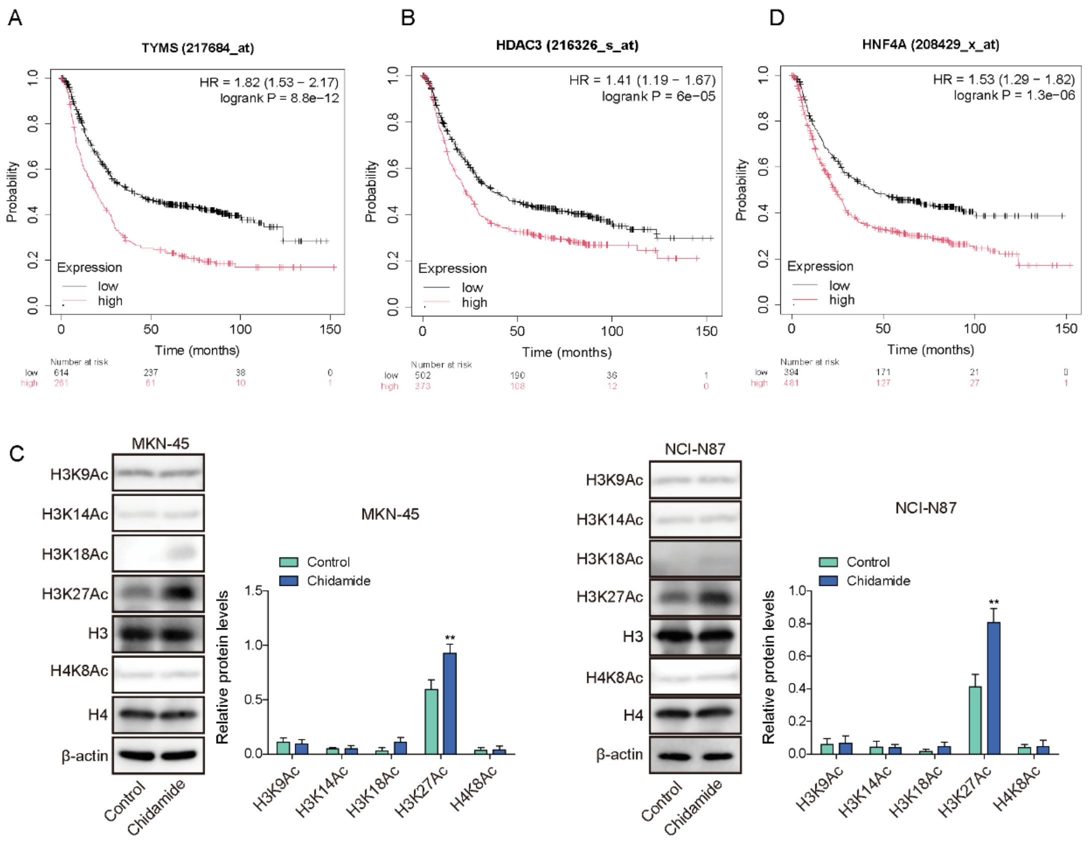

Kaplan–Meier survival analysis of gene expression in gastric cancer using the Kaplan–Meier Plotter database (https://kmplot.com/analysis). (A) TYMS (probe ID: 217684_at), and (B) HDAC3 (216326_s_at). (C) GC cell lines, including MKN-45, HGC-27, were exposed to chidamide at 30 μM for 48 hours. Total protein expression of histones H3 and H4 and the acetylation levels at representative sites were analyzed using Western blot, as were the levels of H3K27Ac, H3K18Ac, H3K9Ac, H3K14Ac and H4K8Ac. (D) Kaplan–Meier survival analysis of HNF4A (208429_x_at)

expression in gastric cancer using the Kaplan–Meier Plotter database. All experiments were repeated at least 3 times, $**p<0.01$.

Supplementary Figure 5. Chidamide promotes the sensitivity of gastric cancer cells to 5-FU by downregulating TYMS.

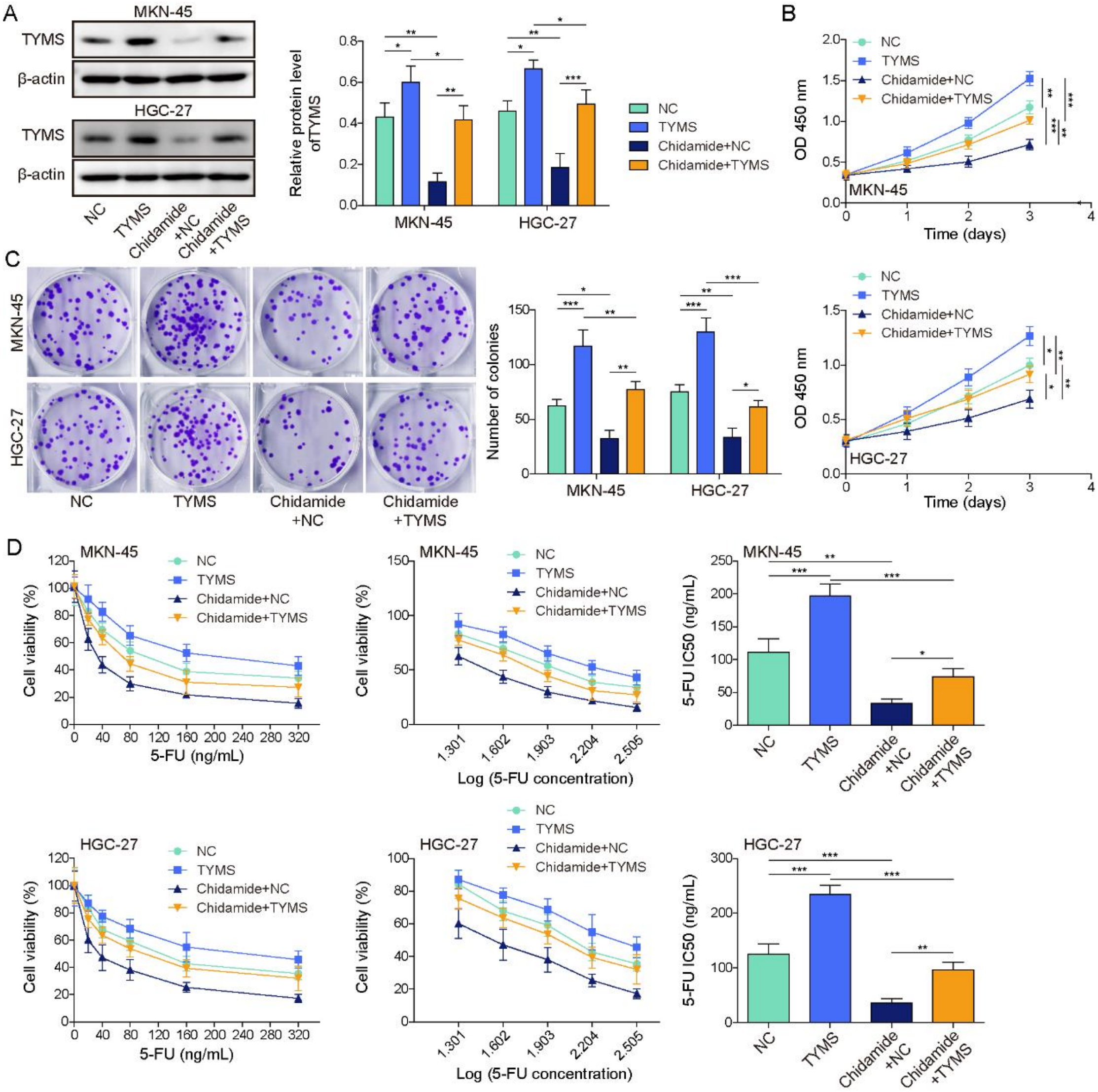

GC cells (MKN-45, HGC-27) were transfected with OE-TYMS or OE-NC and then were treated with chidamide (30 μM). (A) TYMS expression was analyzed using Western blot. (B) Cell viability was measured using the CCK-8 assay. (C) Cell proliferation was assessed via colony formation assay. (D) Sensitivity to 5-FU was

evaluated using the CCK-8 assay. All experiments were repeated at least 3 times, *p<0.05, **p<0.01, ***p<0.001.

Supplementary Figure 6. Chidamide relieved the suppressive effect of HDAC3 on the sensitivity of gastric cancer cells to 5-FU.

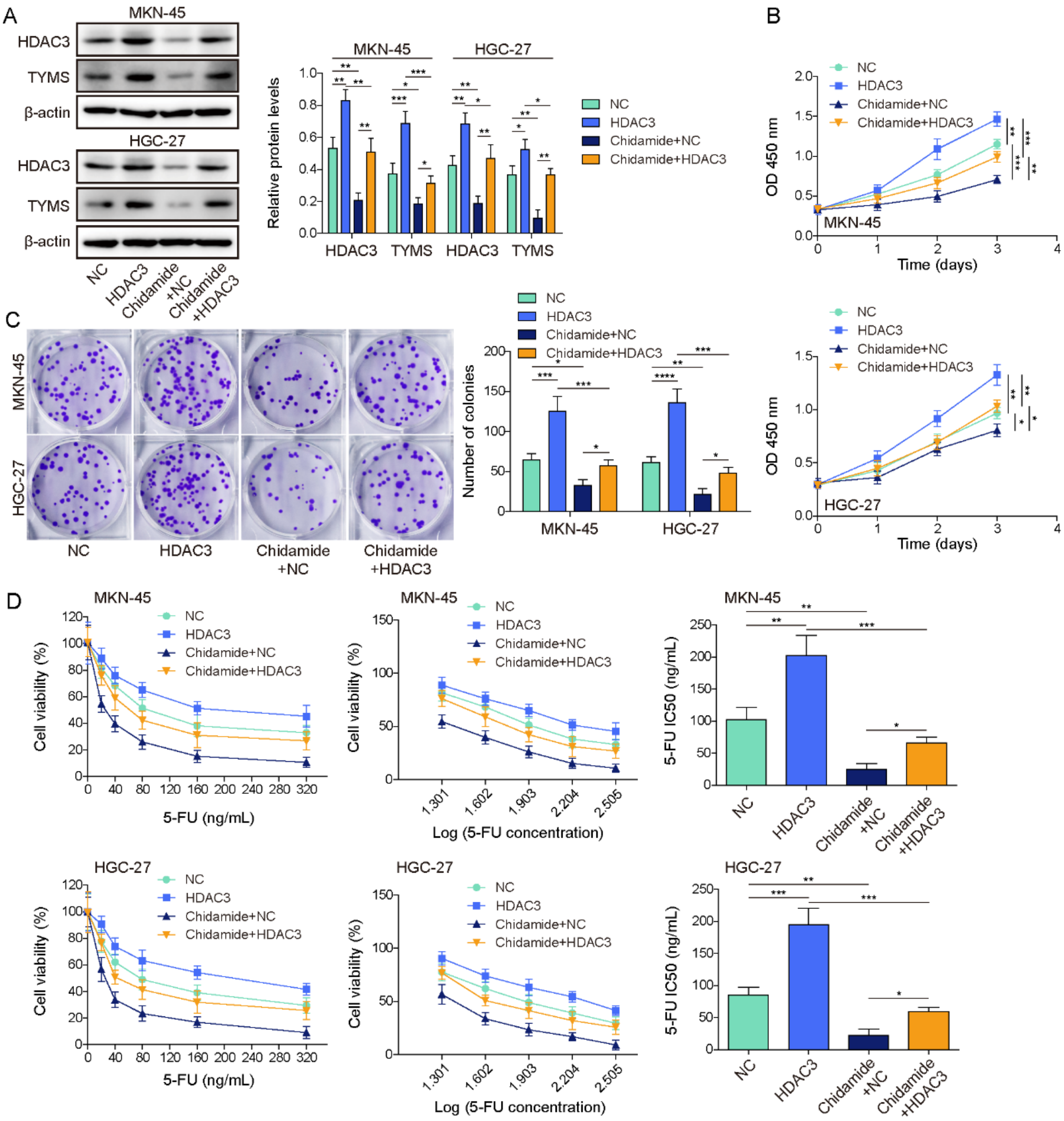

GC cells (MKN-45, HGC-27) were transfected with OE-HDAC3 or OE-NC and then were treated with chidamide (30 μM). (A) TYMS and HDAC3 expression was analyzed using Western blot. (B) Cell viability was measured using the CCK-8 assay. (C) Cell

proliferation was assessed via colony formation assay. (D) Sensitivity to 5-FU was evaluated using the CCK-8 assay. All experiments were repeated at least 3 times, *p<0.05, **p<0.01, ***p<0.001.

Supplementary Figure 7. Chidamide facilitates a marked increase in HNF4A acetylation and a decrease in phosphorylation.

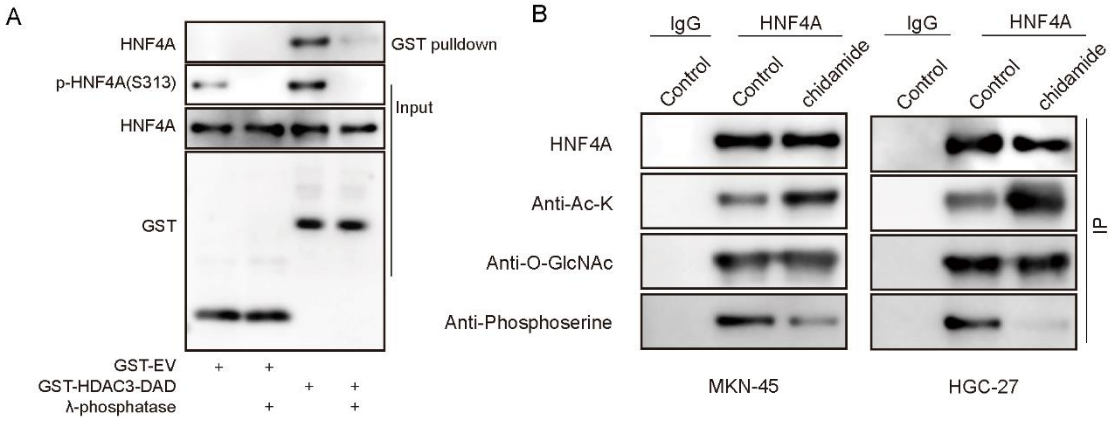

(A) GST pulldown assay was performed to assess the binding between the deacetylase-activating domain (DAD) of HDAC3 and HNF4A in MKN-45 cells. GST-tagged HDAC3-DAD or GST empty vector (GST-EV) was incubated with cell lysates expressing HNF4A. Immunoblotting was conducted using antibodies against HNF4A and phospho-HNF4A (S313). Treatment with λ-phosphatase (500 U, 30 min, 37 °C) was used to reduce phosphorylation levels. (B) MKN45 and HGC-27 cells were treated with or without chidamide. Protein lysates were prepared using RIPA buffer containing protease inhibitors and subjected to immunoprecipitation (IP) with anti-HNF4A antibody. Western blot was performed using antibodies against acetyl-lysine (Ac-K), O-GlcNAc, and phospho-serine to assess HNF4A post-translational modifications. All experiments were repeated at least 3 times.

Supplementary Figure 8. HNF4A K458 acetylation affects S313 phosphorylation, and vice versa.

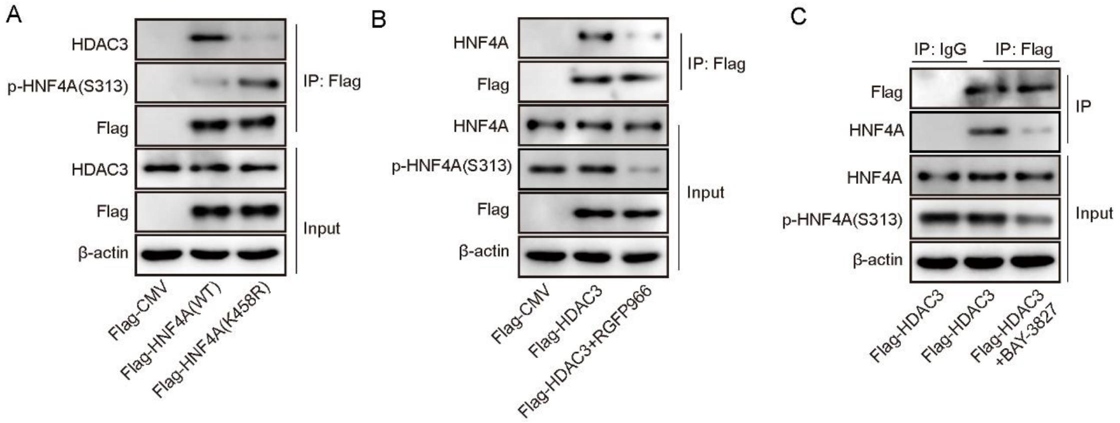

(A) Co-IP analysis of HDAC3 interaction and phospho-HNF4A (S313) in MKN-45 cells transfected with Flag-HNF4A wild-type (WT) or acetylation-deficient mutant (K458R). (B) Co-IP and Western blot analysis of HNF4A and phospho-HNF4A(S313) in MKN-45 cells transfected with Flag-HDAC3 with or without RGFP966 treatment. (C) Co-IP and Western blot analysis of HNF4A phosphorylation and HDAC3 binding in MKN-45 cells transfected with Flag-HDAC3, with or without treatment with the AMPK inhibitor BAY-3827. All experiments were repeated at least 3 times.

Supplementary Figure 9. Chidamide boosted HNF4A acetylation at K458 site.

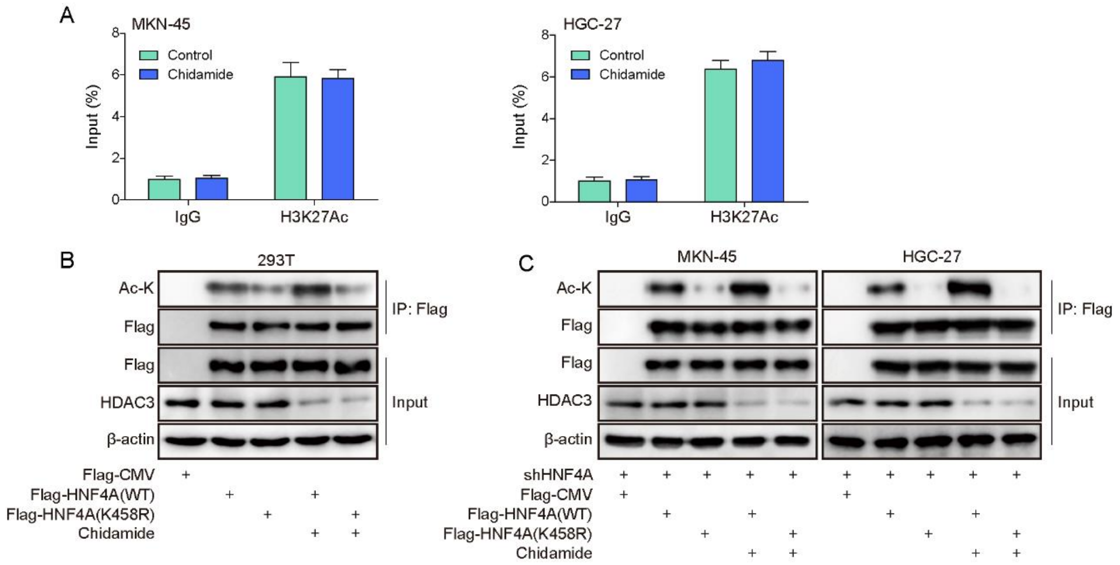

(A) ChIP-qPCR analysis of H3K27Ac enrichment at the TYMS promoter in MKN45 and HGC-27 cells treated with chidamide. (B) Co-immunoprecipitation (Co-IP) analysis of HNF4A acetylation in 293T cells transfected with Flag-HNF4A wild-type (WT) or acetylation-deficient mutant (K458R), with or without chidamide treatment. (C) Co-IP analysis of HNF4A acetylation in MKN45 and HGC-27 cells transfected with Flag-HNF4A WT or K458R, treated with or without chidamide. All experiments were repeated at least 3 times.

2. The full and uncropped Western blot bands

Figure 1

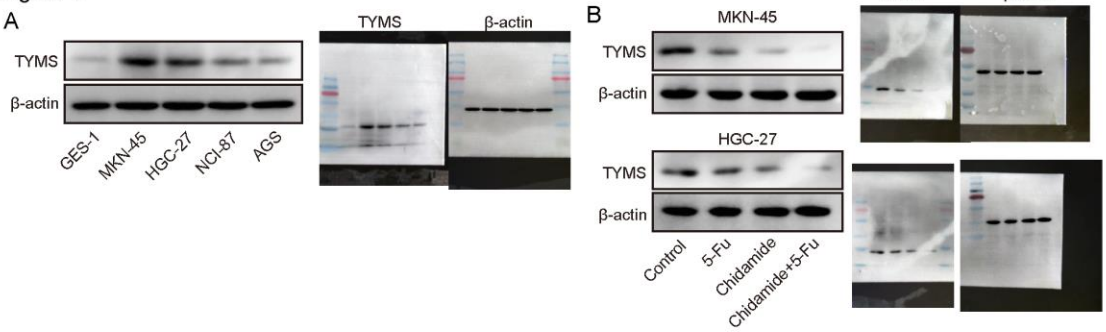

Figure 2

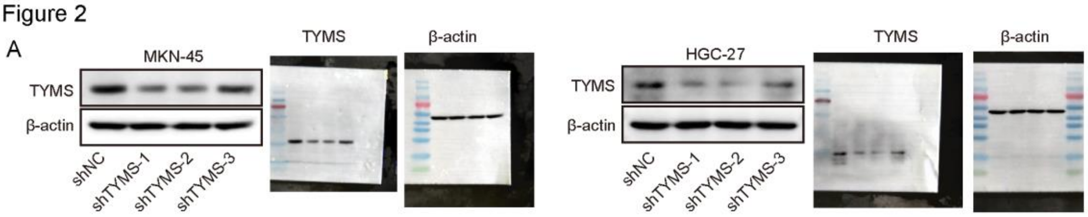

Figure 3

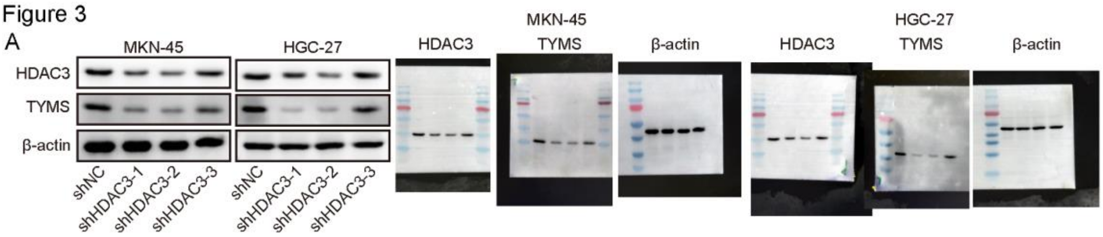

Figure 4

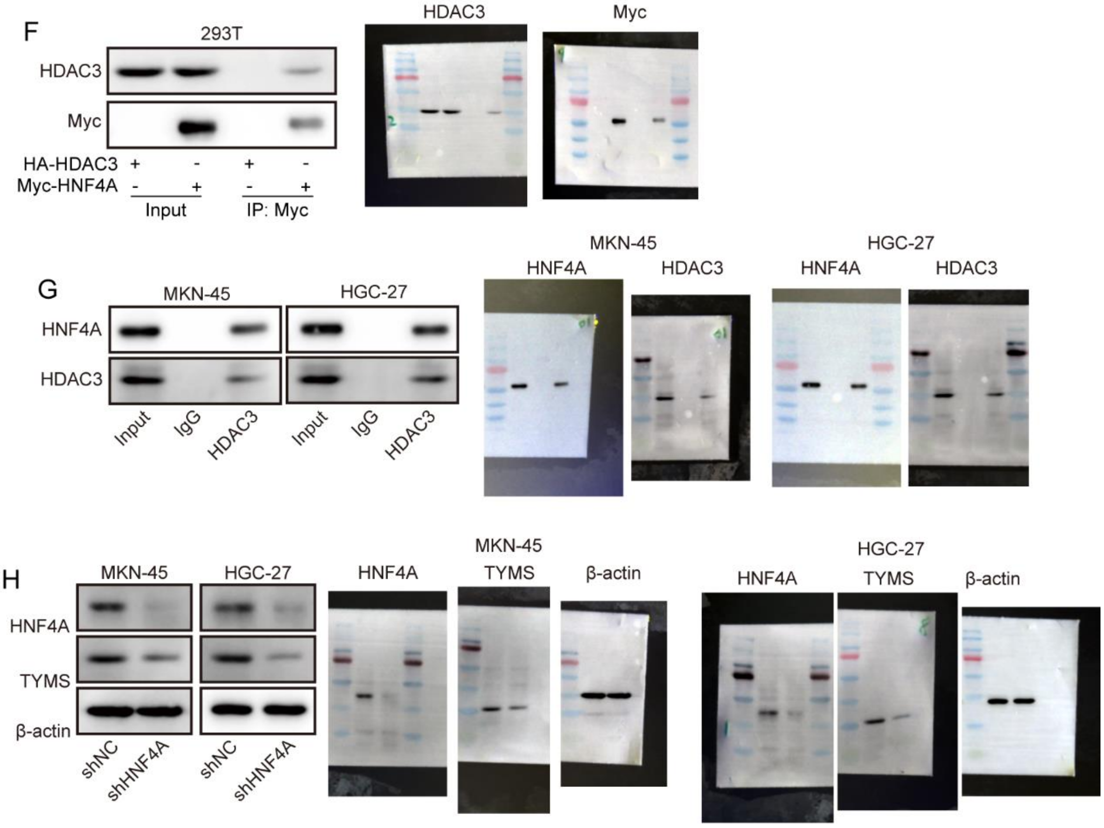

Figure 5

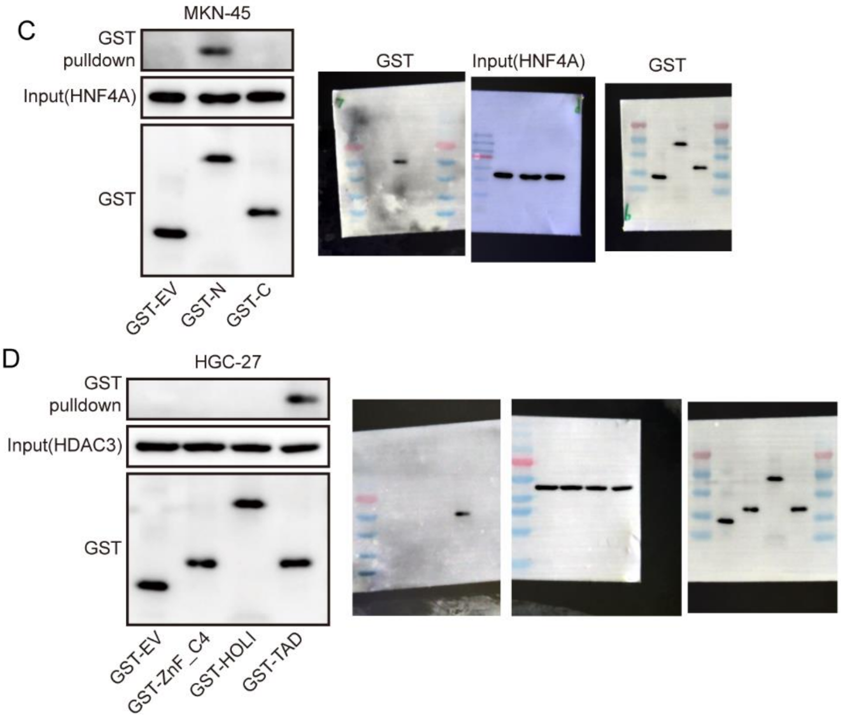

Figure 5

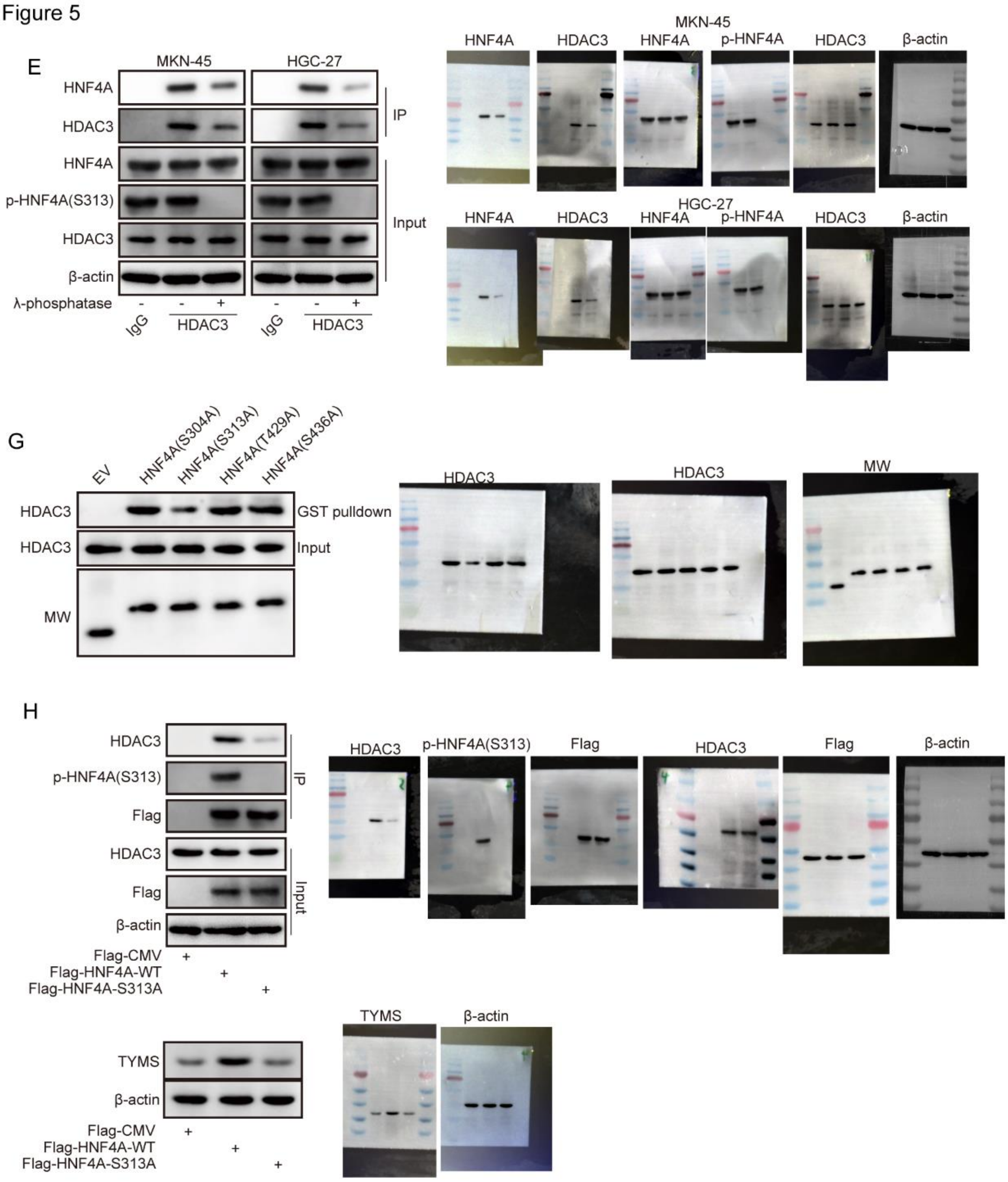
E
MKN-45
HGC-27
HNF4A
HDAC3
HNF4A
p-HNF4A(S313)
HDAC3
β-actin
λ-phosphatase
IgG
HDAC3
IP
Input
p-HNF4A
G
EV
HNF4A(S304A)
HNF4A(S313A)
HNF4A(T429A)
HNF4A(S436A)
GST pulldown
MW
H
Flag
Flag-CMV
Flag-HNF4A-WT
Flag-HNF4A-S313A
p-HNF4A(S313)
TYMS

Figure 5

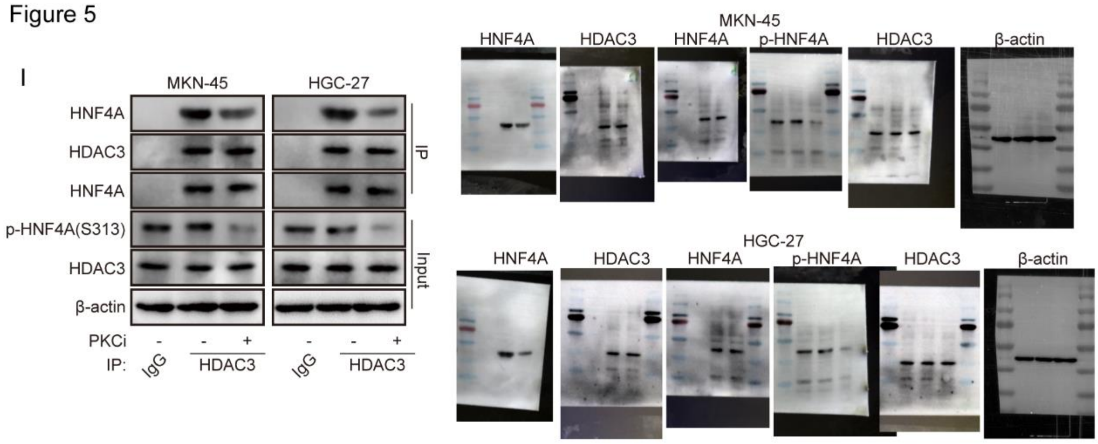

Figure 6

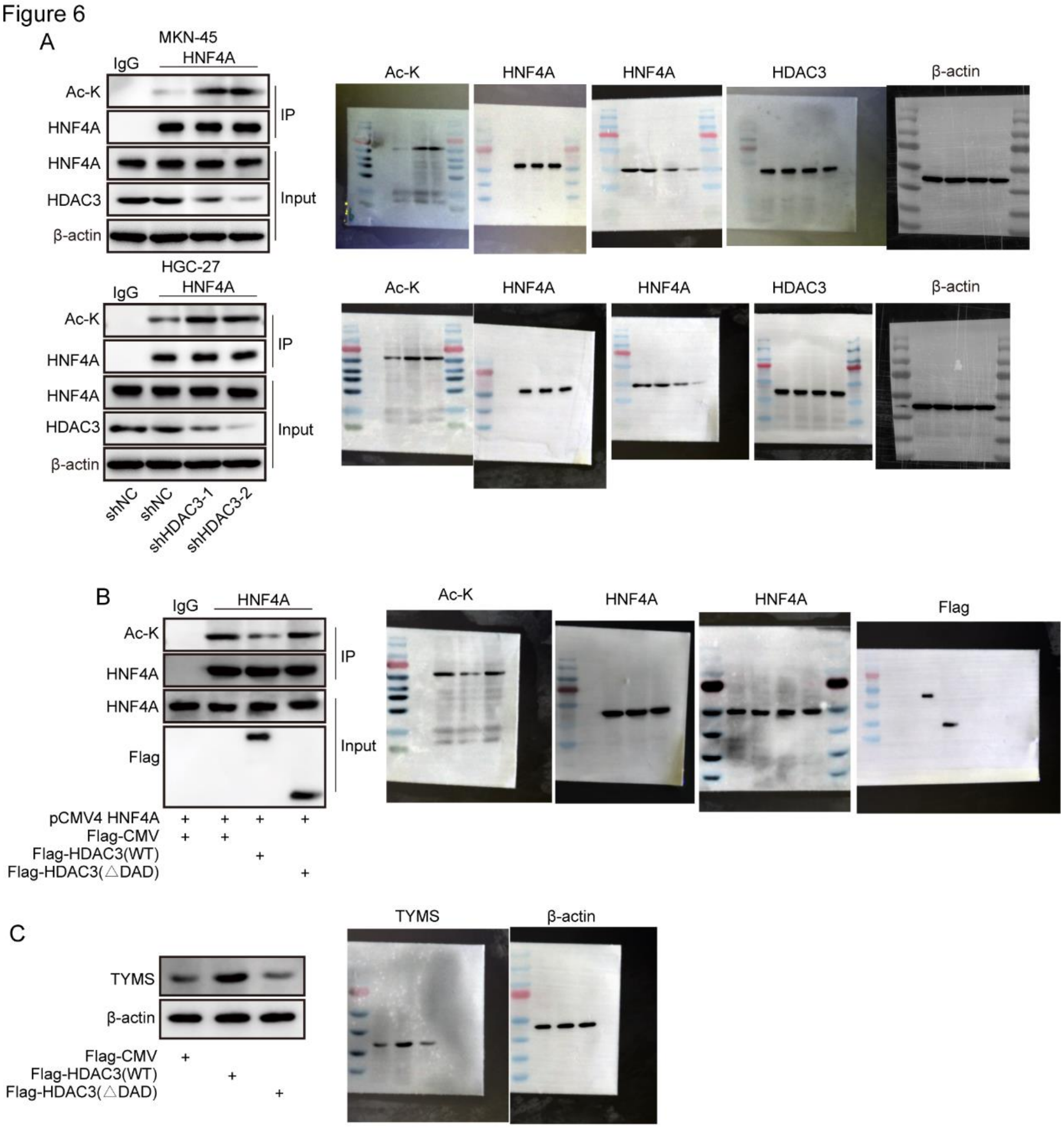

Figure 6

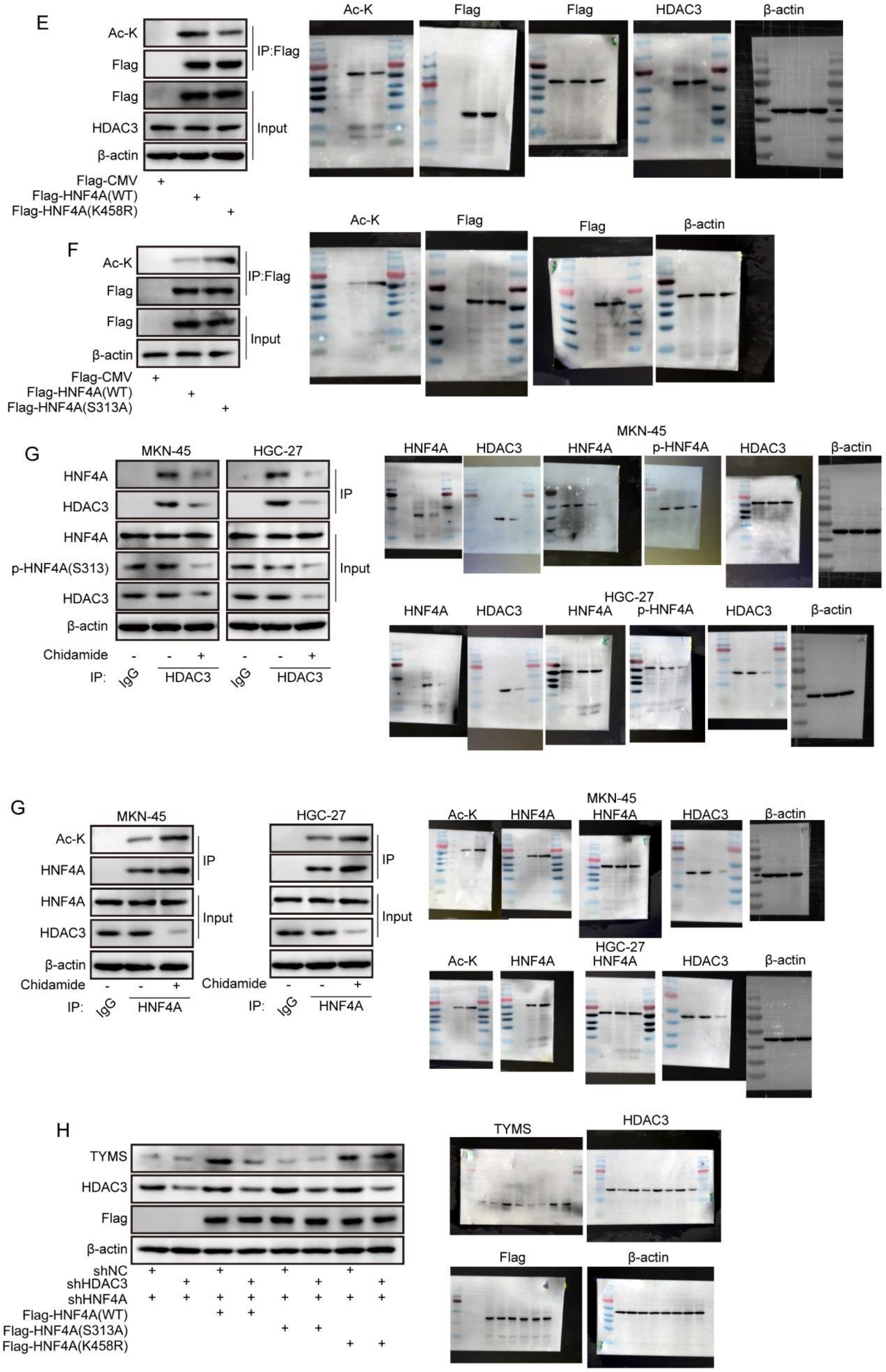

E
Ac-K
Flag
Flag
HDAC3
β-actin
IP:Flag
Input
Flag-CMV
Flag-HNF4A(WT)
Flag-HNF4A(K458R)
Ac-K
Flag
Flag
HDAC3
β-actin
F
Ac-K
Flag
Flag
β-actin
IP:Flag
Input
Flag-CMV
Flag-HNF4A(WT)
Flag-HNF4A(S313A)
Ac-K
Flag
Flag
β-actin
G
MKN-45
HGC-27
HNF4A
HDAC3
HNF4A
p-HNF4A(S313)
HDAC3
β-actin
IP
Input
Chidamide
IP:
IgG
HDAC3
MKN-45
HNF4A
HDAC3
HNF4A
p-HNF4A
HDAC3
β-actin
HGC-27
HNF4A
HDAC3
HNF4A
p-HNF4A
HDAC3
β-actin
G
MKN-45
HGC-27
Ac-K
HNF4A
HNF4A
HDAC3
β-actin
IP
Input
Chidamide
IP:
IgG
HNF4A
MKN-45
Ac-K
HNF4A
HNF4A
HDAC3
β-actin
HGC-27
Ac-K
HNF4A
HNF4A
HDAC3
β-actin
H
TYMS
HDAC3
Flag
β-actin
shNC
shHDAC3
shHNF4A
Flag-HNF4A(WT)
Flag-HNF4A(S313A)
Flag-HNF4A(K458R)
TYMS
HDAC3
Flag
β-actin

Figure 7

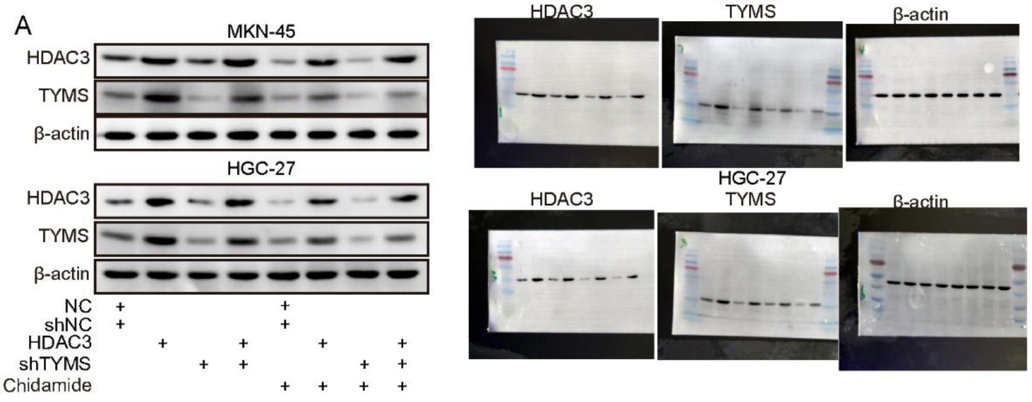

Figure S3

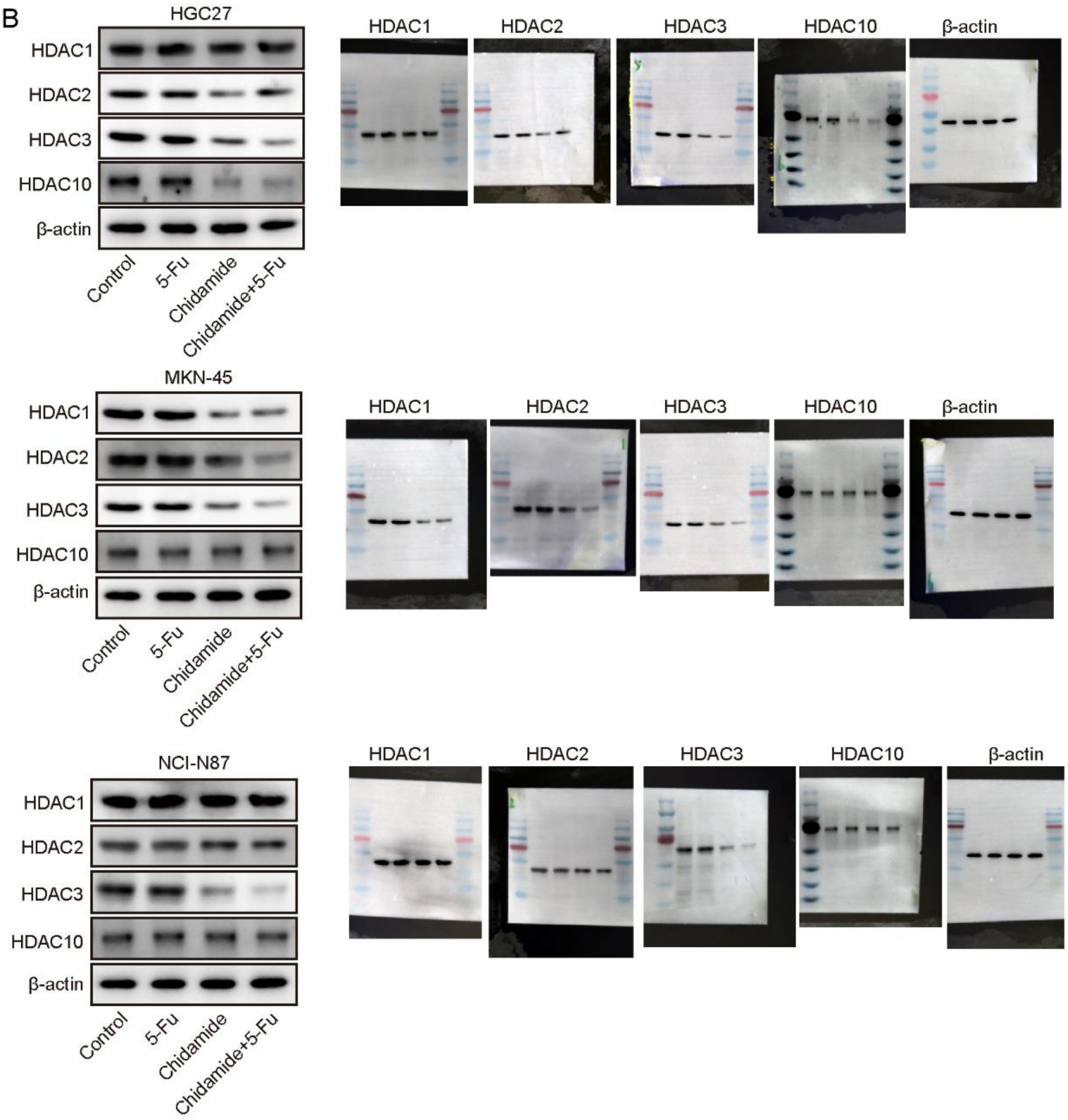

Figure S4

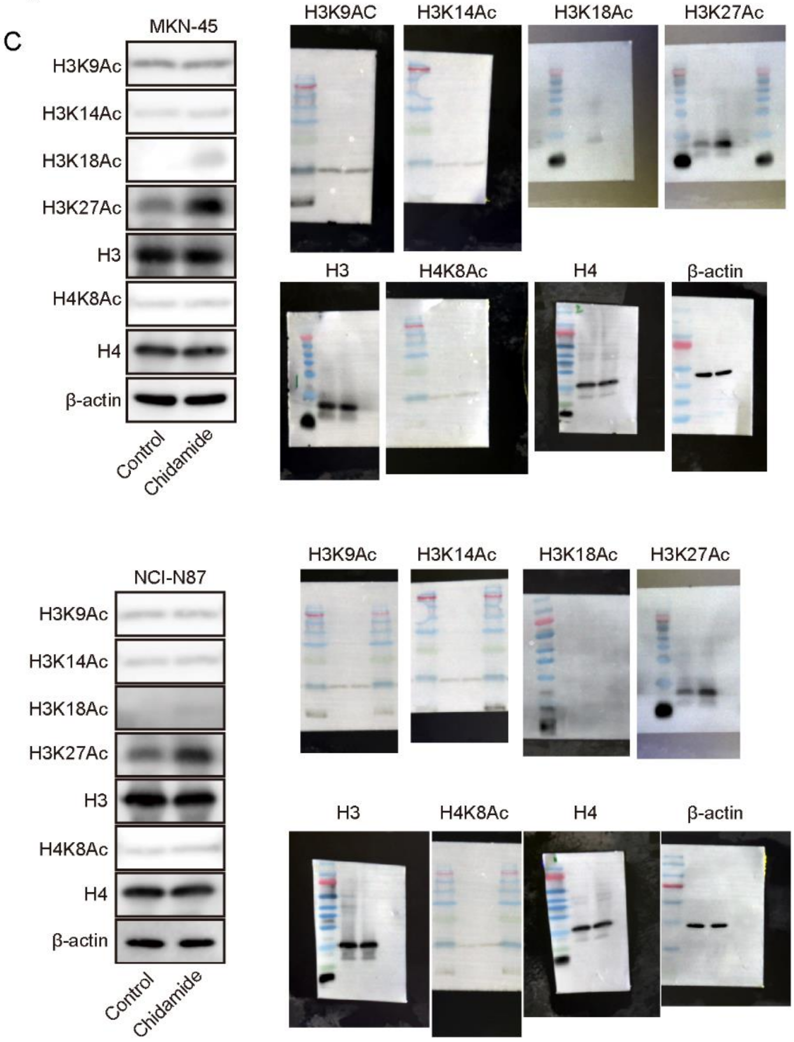

Figure S5

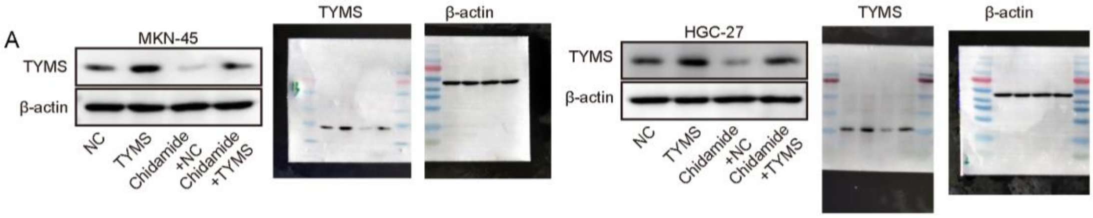

Figure S6

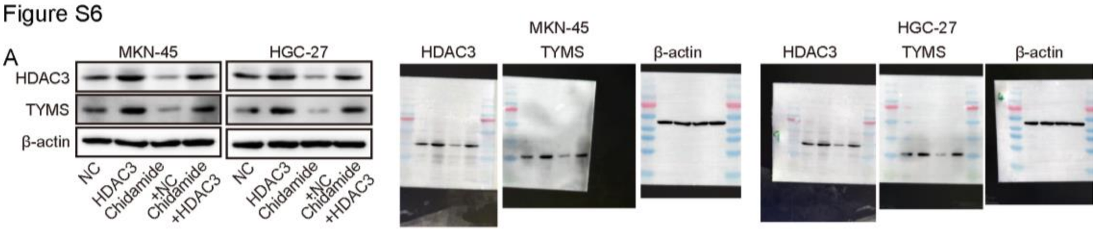

Figure S7

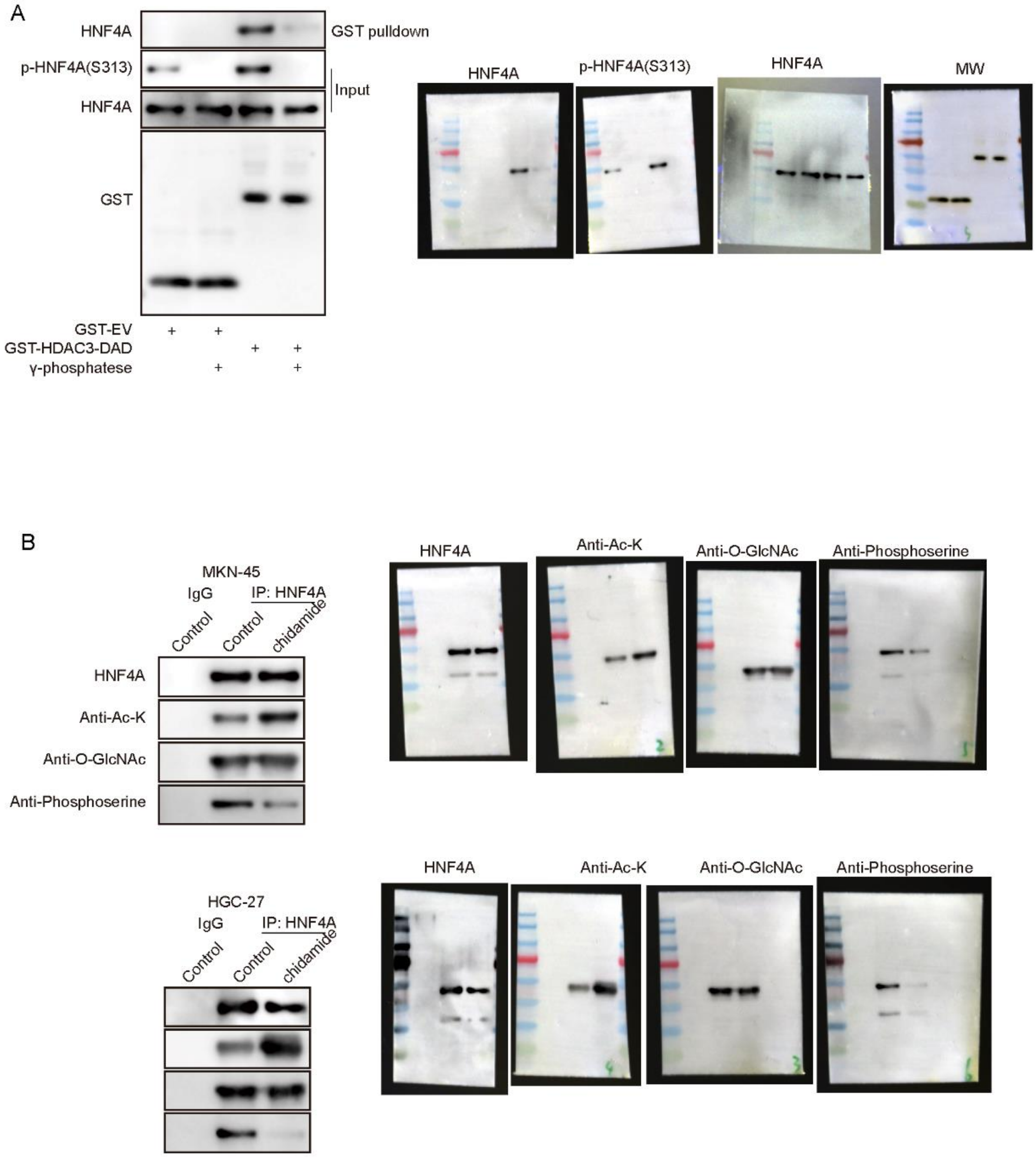
A
HNF4A
GST pulldown
p-HNF4A(S313)
Input
HNF4A
GST
GST-EV
GST-HDAC3-DAD
γ-phosphatese
HNF4A
p-HNF4A(S313)
HNF4A
MW
B
MKN-45
IgG
IP: HNF4A
Control
Control
chidamide
HNF4A
Anti-Ac-K
Anti-O-GlcNAc
Anti-Phosphoserine
HGC-27
IgG
IP: HNF4A
Control
Control
chidamide

Figure S8

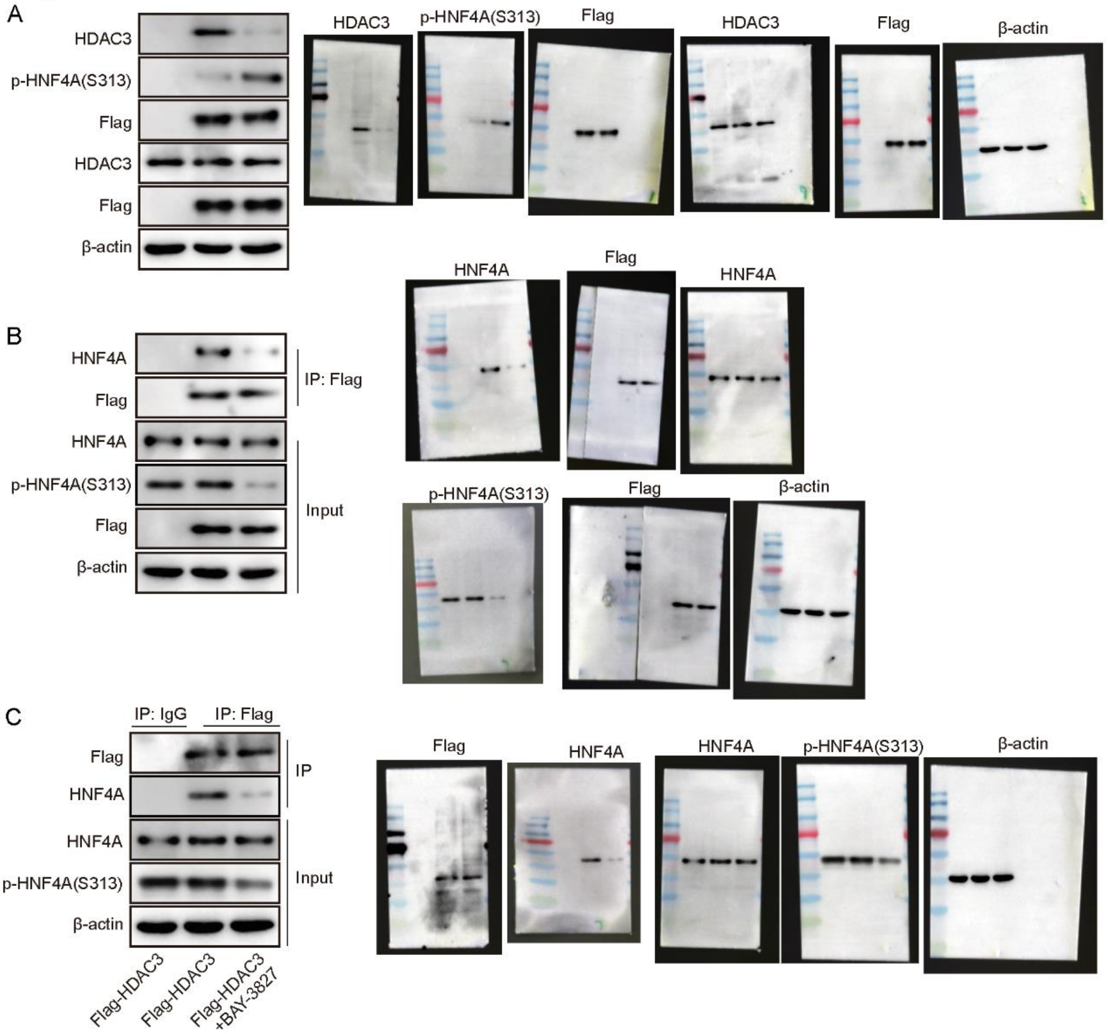

A
HDAC3
p-HNF4A(S313)
Flag
HDAC3
Flag
β-actin
HDAC3
p-HNF4A(S313)
Flag
HDAC3
Flag
β-actin
B
HNF4A
Flag
IP: Flag
HNF4A
p-HNF4A(S313)
Flag
β-actin
Input
HNF4A
Flag
HNF4A
p-HNF4A(S313)
Flag
β-actin
C
IP: IgG
IP: Flag
Flag
HNF4A
IP
HNF4A
p-HNF4A(S313)
β-actin
Input
Flag-HDAC3
Flag-HDAC3
Flag-HDAC3 +BAY-3827
Flag
HNF4A
HNF4A
p-HNF4A(S313)
β-actin

Figure S9

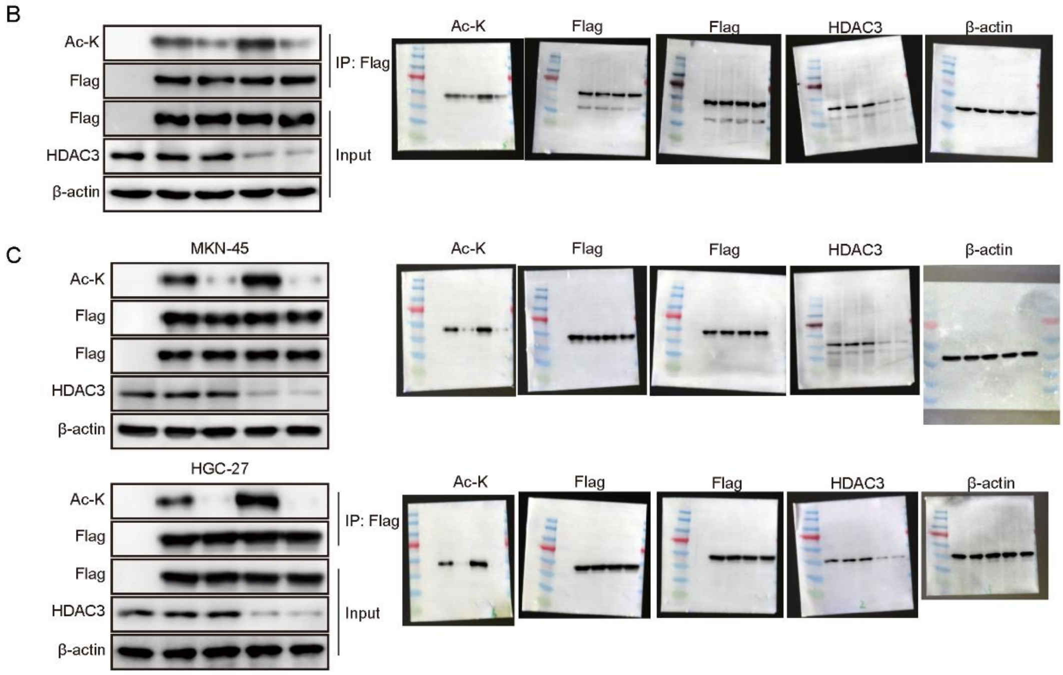
B
Ac-K
Flag
Flag
HDAC3
β-actin
IP: Flag
Input
Ac-K
Flag
Flag
HDAC3
β-actin
C
MKN-45
Ac-K
Flag
Flag
HDAC3
β-actin
Ac-K
Flag
Flag
HDAC3
β-actin
HGC-27
Ac-K
Flag
Flag
HDAC3
β-actin
IP: Flag
Input
Ac-K
Flag
Flag
HDAC3
β-actin
